# Multichannel stimulation module as a tool for animal studies on cortical neural prostheses

**DOI:** 10.3389/fmedt.2022.927581

**Published:** 2022-09-13

**Authors:** Yuki Hayashida, Seiji Kameda, Yuichi Umehira, Shinnosuke Ishikawa, Tetsuya Yagi

**Affiliations:** ^1^Division of Electrical, Electronic and Information Engineering, Graduate School of Engineering, Osaka University, Suita, Japan; ^2^Department of Information Engineering, Graduate School of Engineering, Mie University, Tsu, Japan; ^3^Department of Electrical and Electronic Engineering, School of Engineering, Fukui University of Technology, Fukui, Japan

**Keywords:** animal experiment, multichannel microstimulation, neural prosthesis, neural excitation, neuromorphic device, optical imaging, preclinical study, stimulator device

## Abstract

Intracortical microstimulation to the visual cortex is thought to be a feasible technique for inducing localized phosphenes in patients with acquired blindness, and thereby for visual prosthesis. In order to design effective stimuli for the prosthesis, it is important to elucidate relationships between the spatio-temporal patterns of stimuli and the resulting neural responses and phosphenes through pre-clinical animal studies. However, the physiological basis of effective spatial patterns of the stimuli for the prosthesis has been little investigated in the literature, at least partly because that the previously developed multi-channel stimulation systems were designed specifically for the clinical use. In the present, a 64-channel stimulation module was developed as a scalable tool for animal experiments. The operations of the module were verified by not only dry-bench tests but also physiological animal experiments *in vivo*. The results demonstrated its usefulness for examining the stimulus-response relationships in a quantitative manner, and for inducing the multi-site neural excitations with a multi-electrode array. In addition, this stimulation module could be used to generate spatially patterned stimuli with up to 4,096 channels in a dynamic way, in which the stimulus patterns can be updated at a certain frame rate in accordance with the incoming visual scene. The present study demonstrated that our stimulation module is applicable to the physiological and other future studies in animals on the cortical prostheses.

## Introduction

In previous chronic and acute studies on patients with acquired blindness or those undergoing brain surgeries, conscious perception of small spots of light, commonly known as phosphenes, was artificially elicited by electrical stimulation applied to the surface of the visual cortex (1–8). Since some of these patients could perceive multiple distinct phosphenes in response to the stimuli delivered from spatially separated multiple electrodes, phosphene-based visual prosthesis was expected to be realized (9, 10). However, a critical drawback of this type of stimulation was that the threshold current required to induce the phosphene was in the range of milli-amperes, and that the stimulating electrodes were of relatively large in size (i.e., milli-meters in diameter) for reducing the risk of water electrolysis at the electrode-electrolyte interface during the stimulus current injection (11, 12). These challenges in the cortical-based prosthesis should be overcome, at least partially, by applying intracortical microstimulation because phosphenes were able to be elicited by stimulus current of a few tens of micro-amperes or less using micron-sized electrodes inserted in the visual cortices of humans (13, 14) [see also Button and Putnam (15)] or non-human primates (16). Therefore, the intra-cortical visual prosthesis has been considered promising (17–19) and thought to be applicable to a broad range of acquired blindness conditions (e.g., blindness due to glaucoma, diabetic retinopathy, or dysfunctions in the subcortical visual pathway). In addition, compared with other visual prostheses, it is expected that the largest number of phosphenes may be supplied because of the relatively large tissue area available for the electrode implantation (17, 20). After decades from the detailed clinical experiments on the intracortical visual prosthesis (21), a recent clinical trial verified the feasibility of this type of visual prosthesis (22, 23).

In order to design effective stimuli for the prosthesis, it is thought to be important to elucidate relationships between stimulus parameters and the corresponding properties of neural responses through animal studies. According to previous experiments on the chronaxie estimated for the direct excitation of cortical neurons in cats, the stimulus pulse duration of a few hundred microseconds per phase is considered favorable (1, 24, 25). And, with using such a pulse duration (e.g., 200 μsec/phase), the threshold current required for the cortical neural excitation was in the range of several to a few tens of microamperes in rodents with progressive vision loss (26) as well as normal sight (27, 28). These parameter ranges of the stimulus (i.e., pulse duration and current amplitude) used in the animal studies are in agreement with those for inducing phosphenes by microstimulation in either blind or normal sighted human patients (13, 14, 23) and non-human primates (16, 29). Also, it was reported that the stimulus-induced neural excitations gradually decreased in response to the repetitive pulse stimulation with the frequency of 100–200 Hz in the rodent visual cortex (28). This could explain the clinical and psychophysical observations (30, 31), in which the consciously perceivable phosphenes induced by such a high-frequency stimulation gradually faded in appearance during its relatively long stimulus duration (0.5–1 sec). Based on these lines of results, it is expected that physiological studies in experimental animals provide the basis of effective stimulus parameters applicable to the prostheses.

Previous psychophysical studies with using simulated phosphenes in normal sighted humans suggested that 325–650 dots of phosphenes distributed two-dimensionally in the visual field might be a minimum requirement for achievement of some visually guided tasks (e.g., reading text, eye-hand coordination, and walking in a simplified or virtual maze) (32–35). Accordingly, the electronic device systems that are capable of generating stimuli with spatial patterns from a few tens to several hundred output channels have been developed in previous studies (36–40). Since those systems were specifically designed to be suitable for the clinical purpose, their usability in physiological animal experiments were least demonstrated. Hence, cortical neural responses to spatial patterns of the stimuli with multiple electrodes have been little investigated in the literature. In order to reveal the physiological basis of effective patterns of the stimuli for the prosthesis, a multi-channel stimulation module usable for the animal studies in various scales is required. The objective of the present study is to develop such a stimulation module, and to examine its usability through not only dry-bench tests but also physiological experiments. In addition, we preliminary demonstrate that this module can be integrated into a prototype prosthesis system for future animal studies.

## Multichannel stimulation module

### Design

First, we defined the following as variable parameters of the biphasic current pulse stimulus: the amplitude and duration of each of the cathodic and anodic phases, the temporal order of the phases (i.e., cathodic-first or anodic-first), the inter-phase interval, and the inter-pulse interval. Based on previous studies on the chronaxie (1, 29–31) and the charge threshold for inducing phosphenes (13, 14, 16, 29) or neural excitations (27, 28), the temporal accuracy and the amplitude resolution for controlling those variable parameters of stimulus were roughly determined to be less than several micro-seconds and a few micro-amperes, respectively. Also, we assumed that the pulse repetition frequency of stimulation (i.e., the inverse of the inter-pulse interval) was less than 300 Hz (30, 41). Therefore, if a single pulse of the biphasic current stimulus has the duration of around 0.4 msec, for instance (30), then eight stimulus pulses can be accommodated within such an inter-pulse interval. In addition, the spatial pattern of stimuli should better be updated at 30-50 frames per second (42, 43). These numerical conditions were considered for designing the stimulation module, as described below.

**Figure 1:**
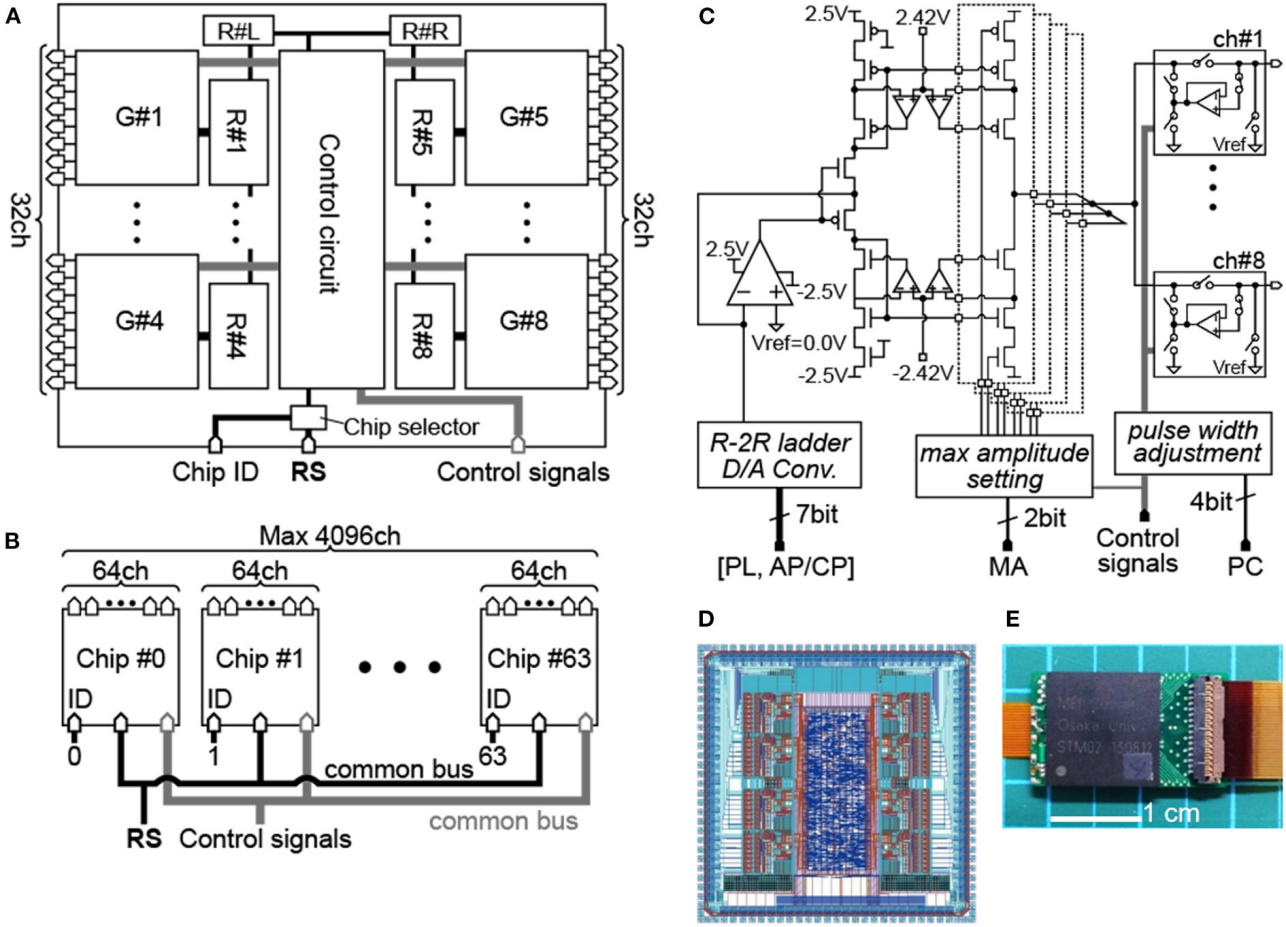
Design of the stimulation module chip. **(A)** Block diagram of the module chip. **(B)** Configuration diagram with the multiple module chips. **(C)** Schematic diagram of the 8-channel current generator. **(D)** Layout design of the module chip. **(E)** Photograph of the packaged chip mounted on a PCB.

Figure 1A shows the main block diagram of our stimulation module as an ASIC chip. Given the chip die size of about 4-by-4 mm, we arranged eight current generators (“G#1” to “G#8” in the figure) together with other circuit blocks in the chip. Based on the above-mentioned consideration of the inter-pulse interval, each of those current generators has eight channels for the stimulus output. Thus, stimulus current pulses can be generated from 8 channels in parallel, or 64 channels at maximum in time-division semi-parallel mode. For use of the time division multiplexing with the 1:8 configuration (i.e., 8 output channels per current generator), the maximum rate of the stimulus pulse repetition in a given output channel is limited by the total length of time required for completing all stimulus outputs from the 8 output channels in a given current generator. The shortest time required for multiplex switching from one to the next output channel is ~7.8 μsec in the present design. Thus, if the time length of every biphasic stimulus pulse is 0.4 msec for instance, then the maximum pulse repetition rate in a given output channel to be ~306 Hz. For controlling the stimulus outputs from the 64 channels, on-chip register memories are implemented (44). Namely, each of the current generators contains a set of data registers (“R#1” to “R#8” in the figure) that stores the stimulation parameters for every output channel, including the current amplitude, the sequential order of output from the channels, and the stimulus on/off switching. In addition, a pair of data registers (“R#L” and “R#R” in the figure) sets the circuit operation parameters, such as maximum current output range, use of the inrush current suppression (described later), and bias voltages in the current generator circuits. Detailed information on these registers, such as hardware locations, register names, data contents, and data sizes, is listed in Table 1. To reduce the overhead time for storing the data into these registers, they are divided into three groups depending on the frequency of data update (see the leftmost column in Table 1). Register group #1 (“Reg1,” 64 bits) manages the stimulus on/off switching and is updated synchronously with the spatial pattern of stimuli. Register group #2 (“Reg2,” 192 bits) is used to set the stimulation sequence and is updated at a moderate frequency. Register group #3 (“Reg3,” 1160 bits) consists of registers for the other stimulus and circuit parameters and is updated infrequently.

**Table 1:** Setting parameters stored to the built-in registers.

**Register**	**Location**	**Name**	**Contents**	**Size**
Reg.1	R#1~#8	STP (G, Ch)	Stimulation position	8G × 8 Ch × 1 bit (on/off) = 64 bits
Reg.2	R#1~#8	STS (G, Ch)	Stimulation sequence	8G ×8 Ch × 3 bits (8 time slots) = 192 bits
Reg.3	R#1~#8	AP (G, Ch)	Anodic phase amplitude	8G ×8 Ch × 6 bits (0~63) = 384 bits
		CP (G, Ch)	Cathodic phase amplitude	8G ×8 Ch × 6 bits (-63~0) = 384 bits
		RP (G, Ch)	Reference channel position	8G ×8 Ch × 1 bits (on/off) = 64 bits
		PA (G, Ch)	Pulse width adjustment	8G ×8 Ch × 4 bits (-7~+8) = 256 bits
	R#L, R#R	MA (S)	Max amplitude	2S (left/right) × 2 bits (4 steps) = 4 bits
		ICS (S)	In-rush current suppression	2S × 2 bits = 4 bits
		OTR (S)	Others	2S × 32 bits = 64 bits

G denotes current generator. Ch denotes output channel. S denotes the side, left or right.

In addition to the register setting (“RS”) signals mentioned above, the digital control signals are also provided from an external device to the module chip *via* bus input ports (“RS” and “Control signals” in Figures 1A,B). Besides, an individual module chip is pre-assigned with an identification (ID) number using a 6-bit digital code set *via* another input port (“Chip ID” in Figure 1A). As illustrated in Figure 1B, multiple module chips (up to 2^6^ = 64) can be connected to the two common buses for the RS and Control signals. The 6-bit ID code is used as the header of the RS signal, and a chip selector circuit in each of the module chip enables updating of the registers when the header is matched with the pre-assigned ID number. After completing the register updates for all of the module chips, the stimulus outputs from all the module chips (up to 64 chips) are driven by the Control signals on the common bus. In other words, the control of a single chip or multiple chips is divided into two phases: initialization and stimulation. In the initialization phase, the data sets in the Reg2 and Reg3 are updated on a chip-by-chip basis. The time required for this phase is 136.2 μsec per chip and thus 8.717 msec for 64 chips at 10 Mbps (17, 45, 46). In the stimulation phase, the data sets in the Reg1 are also updated on a chip-by-chip basis, and then all of the module chips are operated to output the stimulus current pulses by the Control signals in a time-division semi-parallel mode with eight timeslots. The time durations required for updating the Reg1 are 7.3 μsec per chip and 467.2 μsec for 64 chips at 10 Mbps for instance (17, 45, 46). This overhead time is thought to be short enough to control the 64 chips and thus 4,096 stimulating electrodes while updating the stimulation position in the data in the Reg1 (i.e., the spatial patterns of stimuli) every 20 msec (i.e., 50 frame-per-second) for instance (42, 43).

Figure 1C shows the circuit diagram of the current generator, which is composed of an R-2R ladder-type current-mode D/A converter, a differential amplifier, pMOS and nMOS current mirrors, and output circuits (47). In addition, in each of the current mirrors, the gain boosting circuitry with a pair of auxiliary amplifiers is incorporated to realize high output impedance in the voltage range below ±2.42 V. The stimulus current amplitude can be set with a 6-bit code and the maximum output range of the current mirror output is selectable from approximately ±100 to ±400 μA by a ~±100-μA step (“max amplitude setting” in the figure). For instance, considering the threshold stimulus charge for inducing phosphenes or neural excitations (21, 30), the maximum output range of ~±100 μA would be selected as a default. In this range, the amplitude resolution is approximately 1.56 μA (i.e., 100 μA divided by 2^6^). In an actual fabricated chip of the module, however, the amplitude resolution varies between anodic and cathodic currents in a given current generator, and varies also among different current generators (but not among 8 output channels of the same current generator), mostly because of the difference in property of the current mirrors.

The output current flows into or out of either one of eight output circuit blocks (boxes in the rightmost of Figure 1C), each of which is connected to a stimulating electrode at its output port (from “ch#1” to “ch#8”). Thus, by controlling these output circuits, current stimulation is realized by time division with the eight stimulating electrodes. In general, when the surface potential of the electrode in contact with the tissue differs from the potential of the current mirror output, and if the output circuit is a simple analog switch, then an inrush current accompanying the potential difference occurs at the moment when the switch is connected. Therefore, as illustrated in the boxes in Figure 1C, a feedback buffer circuit is added to connect the circuit output and the current mirror output, and the potentials of the current mirror output and the electrode can be rendered equal until the instant before stimulation to suppress the inrush current.

We implemented also a circuit that enables fine adjustment to the pulse width of the second phase in a biphasic stimulus pulse (“pulse-width adjustment” in Figure 1C) for reducing the charge imbalance due to the nMOS/pMOS mismatch. The second phase duration can be either increased or decreased at ~1.95 μsec/step with a 4-bit code in every current generator. In addition, as shown in the boxes in Figure 1C, a switch for shorting of each output channel to a reference electrode/potential (“Vref” in the figure) in the tissue is also built into the output circuit such that charges accumulated at the electrode surface and in the tissue, as well as the residual DC current (48), can be further reduced through such a shorted connection. In practice, the amplitudes and the durations of both anodic and cathodic phases are first set, and then the pulse width adjustment would be used to minimize the charge imbalance for every current generator, while measuring the output current in a dry-bench test and/or a wet-bench test prior to an animal experiment. For certain animal experiments, this procedure can be enough for determining the stimulus charge with the best possible accuracy. However, the balancing of charge only with this procedure may not be perfect, and gradual charge accumulation might occur due to unpredictable factors in biological tissues and the electrode-tissue interface during a long-term stimulation in animals *in vivo*. In such a case, the shorting to Vref can reduce the charge accumulation to compensate for the imperfectness of the above-mentioned procedure. At least in some animal experiments, these two approaches, i.e., fine pulse-width adjustment and output shorting, suffice for suppression of the voltage accumulation during repetitive pulsing of the stimulus current (12), and may offer an alternative to attaining a perfect charge balance of every single stimulus pulse (49–54). Besides, these circuit operations are optional, allowing the user to intentionally test the possible effects of charge imbalance on neural responses or electrode/tissue damages in experimental animals.

Figure 1D shows the layout design of the module chip with a die size of 3.64 mm by 3.64 mm, in which the control circuit at the center and the eight current generators at the right and left sides are recognizable. This chip was fabricated *via* the 0.25-μm CMOS process (Taiwan Semiconductor Manufacturing Co., Ltd), packaged and mounted on a printed circuit board (PCB) (Figure 1E).

### Dry-bench test

Basic operations of the module were bench tested. In those tests, the 6-bit high/low voltage code for the “Chip ID” was supplied from the power line on the PCB. The RS and Control signals fed to the chip were generated by either one of a portable pattern generator (UPG-2116, Japan Data Systems Inc., Hyogo, Japan), an FPGA integration module (Spartan-6, Xilinx, CA, U.S.A.; XEM6010-LX150, Opal Kelly Inc., OR, USA), or a prototype wireless module system (55). Some examples of the results of dry-bench tests are described below.

First, the power consumption of the module chip was measured. The SPICE simulation with the present chip design produced an estimated power consumption of ~2.54 mW without stimulus output, and an actual power consumption measured in the dry-bench test was ~2.27 mW. When the number of the output channel in use was increased under the condition that the biphasic current pulses (~40 μA/phase in amplitude, 0.1 msec/phase in duration, 200 Hz in frequency) were passed for every output channel to a ~10-kΩ resistor, the actual power consumption increased by ~21 μW per channel. These actual power consumptions changed little (<0.5 %) when the clock frequency for generating the digital RS and Control signals was varied in the range of 1–12 MHz.

**Figure 2:**
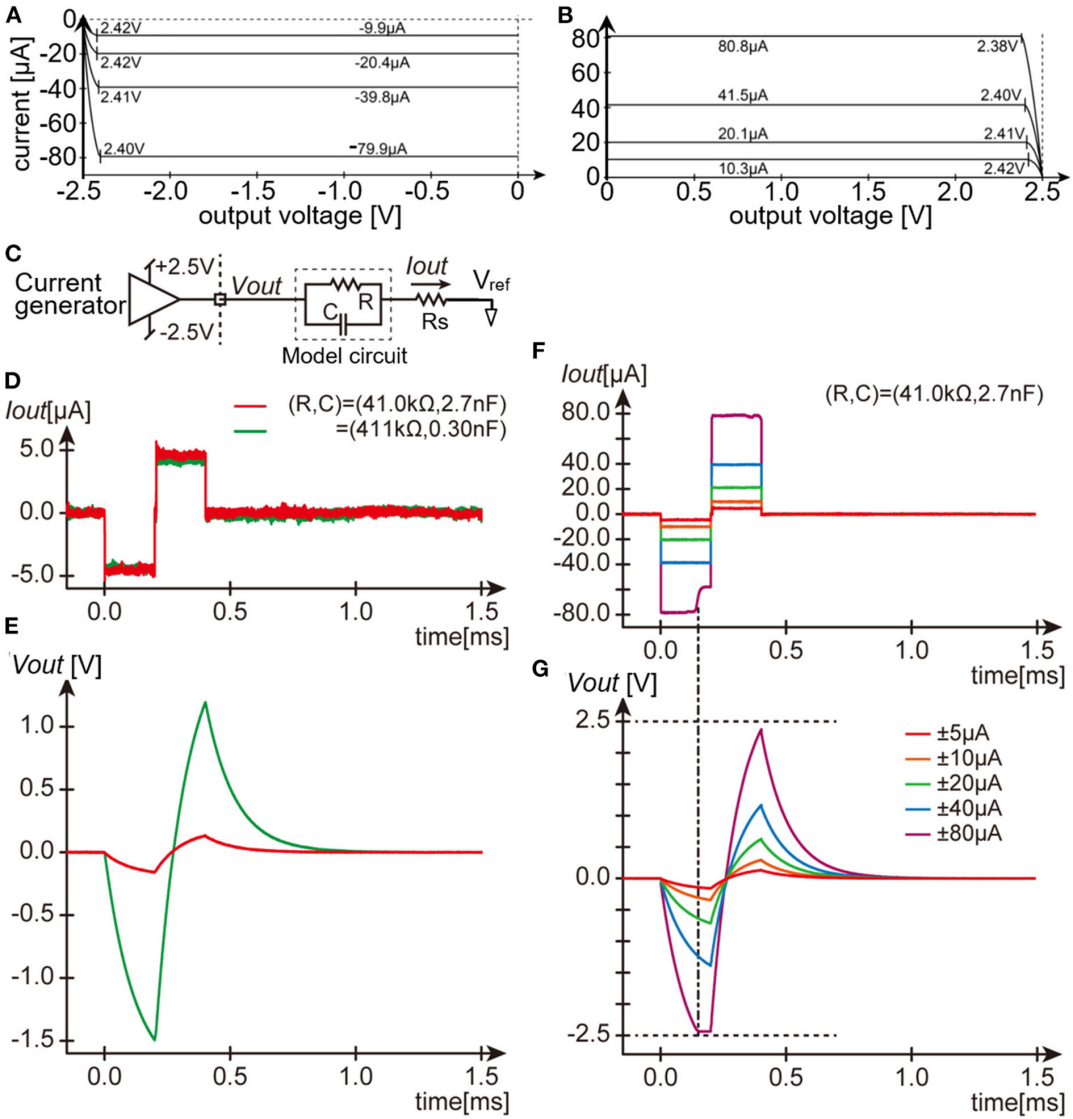
Dry-bench tests of the stimulation module. **(A,B)** Measurements of the output DC impedance of the current generator for the negative current **(A)** and positive current **(B)**. **(C)** Schematic circuit diagram testing the current pulse output. **(D,E)** Recorded traces of the output current pulses **(D)** and the resulting output voltage **(E)** with the same amplitude setting but with two different parameters of the model circuit. **(F,G)** Recorded traces of the output current pulses **(F)** and the resulting output voltage **(G)** with different amplitude settings and with the same model circuit parameters. Note the voltage drop across Rs (~80 mV in the largest case) is hardly visible in the traces in **(E**,**G)**.

Second, the output DC impedance of the current generator in the module was examined. An Ampere meter (ammeter) and a voltage source (34405A and E3631A, Agilent Technologies Inc., CA, USA.) were connected in series to one of the output channels of the module. The voltage at the channel output was varied from zero to either +2.5 or −2.5 V by the voltage source while the command current amplitude of the current generator in the stimulation module was set to a constant value of either one of approximately ±10, ±20, ± 40, and ±80 μA, and the actual output current was measured by the ammeter. Figures 2A,B plots the measured output current against the voltage at the channel output. In either the anodic (A) or the cathodic (B) direction, the output current level remains constant when the output voltage is less than approximately ±2.4 volts, which is the limit voltage determined by the gain boosting circuitry in the current generator (see Figure 1C). The output current level varies less than 0.1 μA (the resolution of the ammeter used) in total when the output voltage level varies from 0 to ~±2.4 volts, indicating that the output DC impedance is higher than 24 MΩ. This was the case in all the 8 current generators, and hence all the 64 output channels.

Third, the pulse output from the current generator in the module was examined with using practical impedance loads. The parallel circuit of a resistor and a capacitor was used as a model of the electrode-electrolyte interface (11), and was connected in series with a ~1-kΩ resistor (“Rs”) to one of the output channels of the module (Figure 2C). Two model circuits were used in this test example; one mimicked a metal-based electrode usable for intracortical stimulation (16) (~33.7 kΩ at 1 kHz), and another mimicked a similar electrode but with much smaller surface area (~325 kΩ at 1 kHz). Figure 2D shows the current traces recorded as voltage drops across the resistor Rs (“*Iout*” in Figure 2C) and Figure 2E shows the resulting voltages recorded with respect to the voltage reference (“*Vout*” in Figure 2C). The waveforms of the current pulses are nearly the same (red and green traces in D) regardless of the difference in voltage drop (red and green traces in E) between the two model circuits. As expected, when the output voltage exceeds the limit voltage of ~±2.4 V, the current output from the generator is limited (Figures 2F,G). Namely, at the amplitude setting of ~±80 μA, the current amplitude during the cathodic phase was first kept near the command value, but subsequently decreased to a smaller value as the output voltage reached the limit voltage. The similar tests were conducted with using other output load impedance ranging from ~8 to ~513 kΩ at 1 kHz. The 10–90% rise time was equal to or shorter than 5 μsec, and the current amplitude during the cathodic and anodic phases were fluctuated by less than 10 %, as far as the output voltage were below the limit value and as the output impedance was lower than around ~150 kΩ at 1 kHz (not shown). We also verified that the current outputs greater than ±100 μA/phase in amplitude were able to be generated when a 1-kΩ resistor was connected as the output load (Supplementary Figure 1).

**Figure 3:**
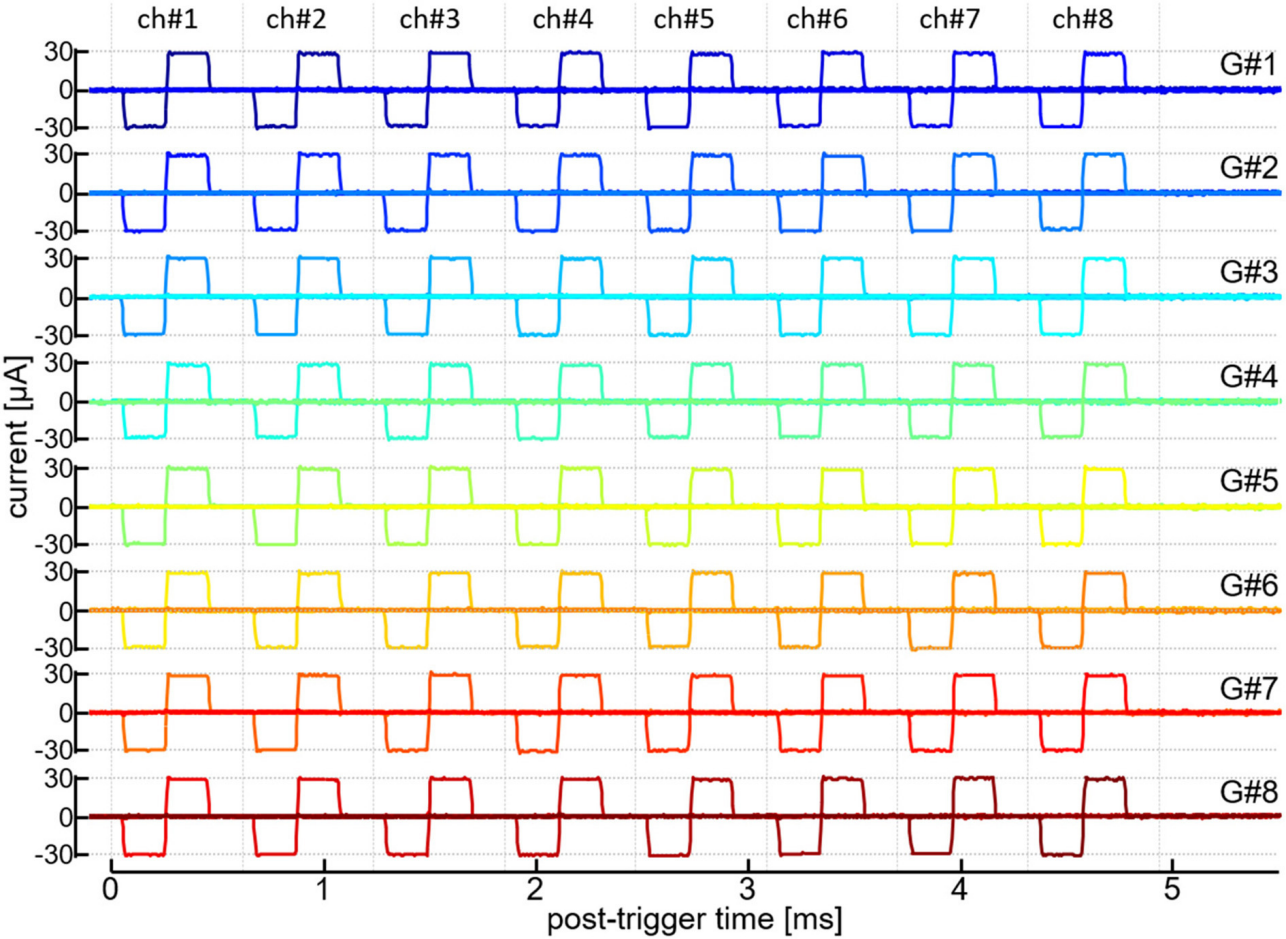
Time-division semi-parallel mode of stimulus outputs. Recorded traces of the output current pulses from 64 output channels are shown on the same timeline axis. Labels in the rightmost, “G#1” to “G#8,” indicate the current generators in operation. Labels in the uppermost, “ch#1” to “ch#8,” indicate the output channels of every current generator.

Fourth, we verified that the current pulse outputs from all the 64 output channels of the module can be operated in a time-division semi-parallel manner. A ~10-kΩ resister was connected to every output channel and the current output was recorded as voltage drop across the resistor. Figure 3 shows the traces of those current outputs from all the 64 channels. In this recording, a time window of ~4,936 μsec was divided into 8 timeslots and, in each of those time slots, every current generator (from “G#1” to “G#8”) generated the biphasic current pulse with one out of the 8 output channels (from “ch#1” to “ch#8”). The register setting values for the anodic and cathodic amplitudes (i.e., AP and CP in Table 1) of every current generator were selected so that the actual amplitudes in all the 64 output channels were approximately ±30 μA. In the figure, each row overlays 8 traces of the current outputs from the 8 output channels of a particular current generator, and the 64 traces on the same timeline are shown. The onset and offset timings as well as the amplitudes of these current pulses little fluctuated when the same pattern of the outputs were repeated, showing the reliable operation of the time-division semi-parallel mode. Also, the time precision as well as the current amplitude accuracy were little affected by varying the number of the generators/output channels in use (not shown).

## Physiological experiments

Our previous studies demonstrated the usefulness of the voltage-sensitive dye (VSD) imaging technique for measurements of the spatio-temporal neural responses to single-site microstimulation in the visual cortex of rodents *in vivo* (26–28, 56). We utilized this technique to examine the practical usability of the stimulation module for multi-site neural excitations in the cortex.

### Methods

**Figure 4:**
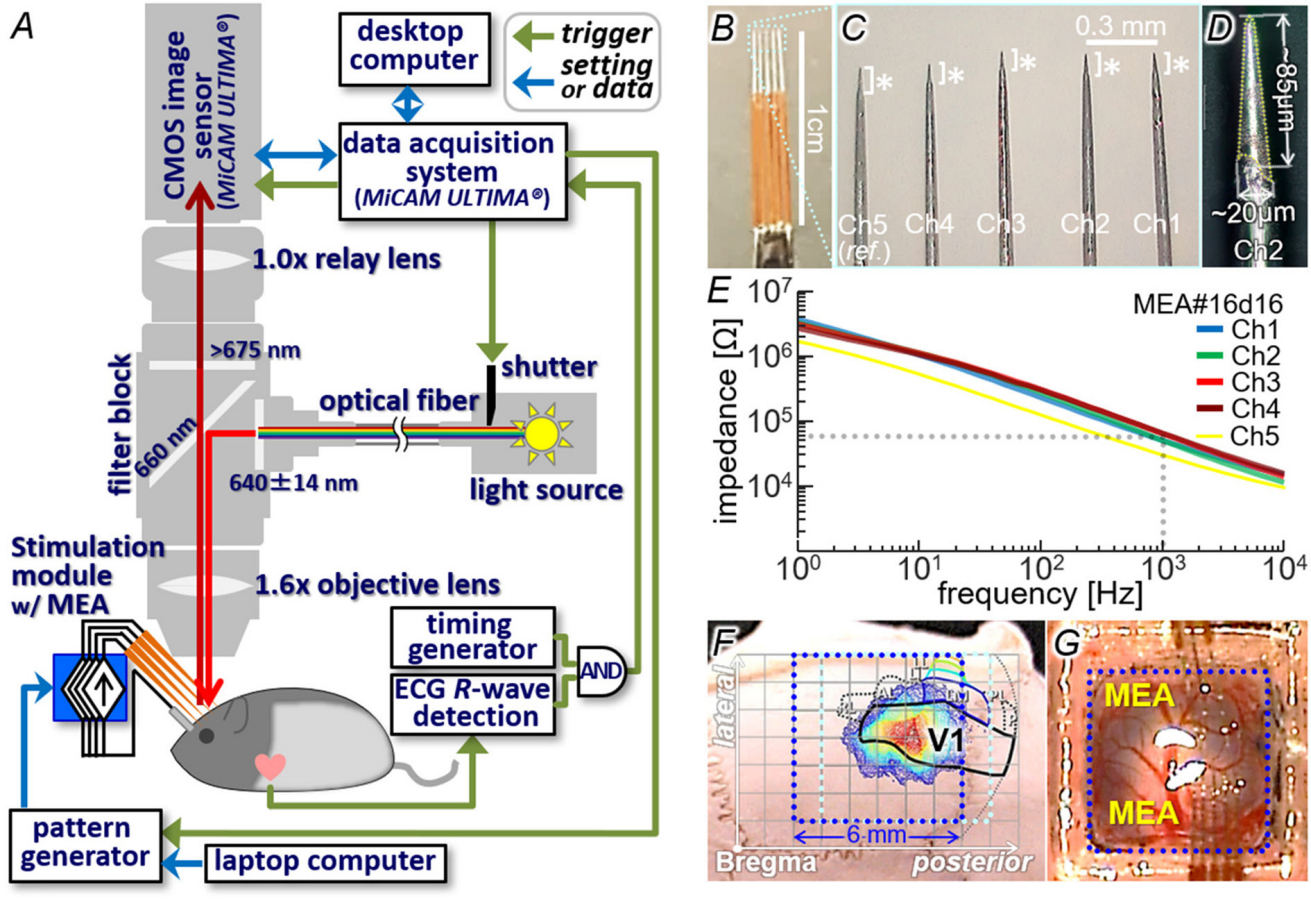
Animal experiment setup. **(A)** Schematic diagram of the voltage-sensitive dye imaging system combined with the stimulation module. **(B–D)** An example of multi-electrode array **(**MEA**)** used for the intracortical stimulation. This MEA was used in the experiments shown in Figures 7–9. **(E)** Measured impedance profiles of five electrodes shown in **(C)**. **(F)** Location of the craniotomy on the skull with overlying the light-induced response profile and the visual cortices atlas. **(G)** Exposed cortex stained with VSD and two penetrating MEAs.

All animal care and experimental procedures in the present study were approved by the Animal Experiments Committee of Osaka University and conducted in conformity with the ‘Guidelines for Proper Conduct of Animal Experiments' by the Science Council of Japan. The Long-Evans rats (24 rats, 6 to 16 weeks old, male and female; Japan SLC Inc., Shizuoka, Japan) were used because of their enough area of the visual cortex for insertion of a multi-electrode array (MEA) (Figure 4G). During the surgery and the imaging experiment, the animal's rectal temperature was feedback-controlled at ~37 °C and electrocardiogram (ECG) was monitored. The surgical procedures and the dye staining were almost the same as those in the previous experiments in rats *in vivo* (26, 55). In brief, following administration of atropine (10 μg) and dexamethasone (20 μg), anesthesia was made with either (1) intraperitoneal (IP) injection of a mixture of medetomidine (0.15 mg/kg-b.w.), midazolam (2 mg/kg-b.w.) and butorphanol tartrate (2.5 mg/kg-b.w.), (2) IP injection of urethane (5 ml/kg-b.w. with 25 wt-% soln., or (3) isoflurane inhalation (0.5–4 % at ~0.5–2 l/min). Craniotomy was performed on the right posterior parietal bone (2–8 or 3–9 mm posterior and 1–7 mm lateral from the Bregma; blue and light blue dotted boxes in Figure 4F) and a custom-made chamber was attached on the bone around the craniotomy. The exposed cortex with the dura intact or removed partially was stained with VSD (RH1691 or RH2080; Optical Imaging, Ltd, Rehovot, Israel). The dye staining of layer II/III (57) was confirmed in a coronal section of the cerebrum after the imaging experiment.

Figure 4A illustrates the imaging setup (26, 55) combined with the stimulation module. Similar to the dry-bench test, the RS and Control signals fed to the stimulation module were generated by either the portable pattern generator or the prototype wireless system (see Section Dry-bench test). A custom-designed MEA (MicroProbes, Inc., MD, USA; Figures 4B–D), in which three to five needle-shaped iridium electrodes were aligned in line, was connected to the output channels of the stimulation module. In the example shown in the figure, the inter-tip distances were ~0.29–0.36 mm (C) and the electrically-exposed tips (asterisks in C and area with dotted outline in D) had conical shapes with base diameters of ~20 μm and lengths of roughly 100 μm (D). Before the experiment, the exposed tip of each electrode was activated so that the impedance was reduced to ~10–65 kΩ at 1 kHz as measured in an artificial cerebrospinal fluid (Figure 4E). The operations of the stimulation module and the imaging system were controlled with using a common trigger signal generated with reference to the ECG R-wave (arrows of “*trigger*” in Figure 4A). In some experiments, a commercially available desktop stimulator (STG2008 with the ±1.6-mA current range, MultiChannel Systems, GmbH, Germany) was also used for comparison. The imaging method was the same as that in the previous experiments (26, 55). In brief, the VSD fluorescence emitted from a 6.25 × 6.25-mm area on the cortex under the excitation illumination was captured at 1000 frames/s with a 100 × 100-pixel CMOS sensor (MiCAM-Ultima, BrainVision, Inc., Tokyo, Japan). The captured image stream was processed as described previously (26, 27) so that the pixel values of an image represent the fractional fluorescence changes induced by the stimulus (referred to as Δ*F/F* in the following text). In the Δ*F/F* image stream, the increase and decrease of pixel value correspond to relative depolarization and hyperpolarization, respectively, of the cytoplasmic membrane.

In order to identify the area of the primary visual cortex (V1), diffuse light stimulation (~13–16 photon/μm^2^/msec in intensity and 20–500 msec in duration; 525-nm LED as the source) was applied to the left eye *via* a light guide. At 20–40 msec after the stimulus onset, an increase in Δ*F/F* was initiated in a certain cortical area (color contour plot in Figure 4; also **Figures 6B**, **7B**), the location of which agreed with the V1 location on the rat brain atlas (58, 59) (black outline in Figure 4F). Through the craniotomy window, the electrode tip was inserted in the V1 area to reach a depth of 300–600 μm from the surface (Figure 4G). The insertion angle was approximately 40° to the cortical surface plane. With this configuration, the cortical surface was only slightly obscured from sight by the electrode shafts of MEA under the epifluorescence microscope (see **Figures 6A**, **7A**, **9A**). A pellet or winding wire of Ag/AgCl was placed as the return electrode for stimulation in the head chamber, or in the mouth in a few experiments. The return electrode was connected the voltage reference port of the stimulation module or of the desk-top stimulator. The current pulse stimuli were delivered at inter-stimulus intervals of more than 10 sec to avoid any irreversible effects on the tissue and/or the response.

### Results

**Figure 5:**
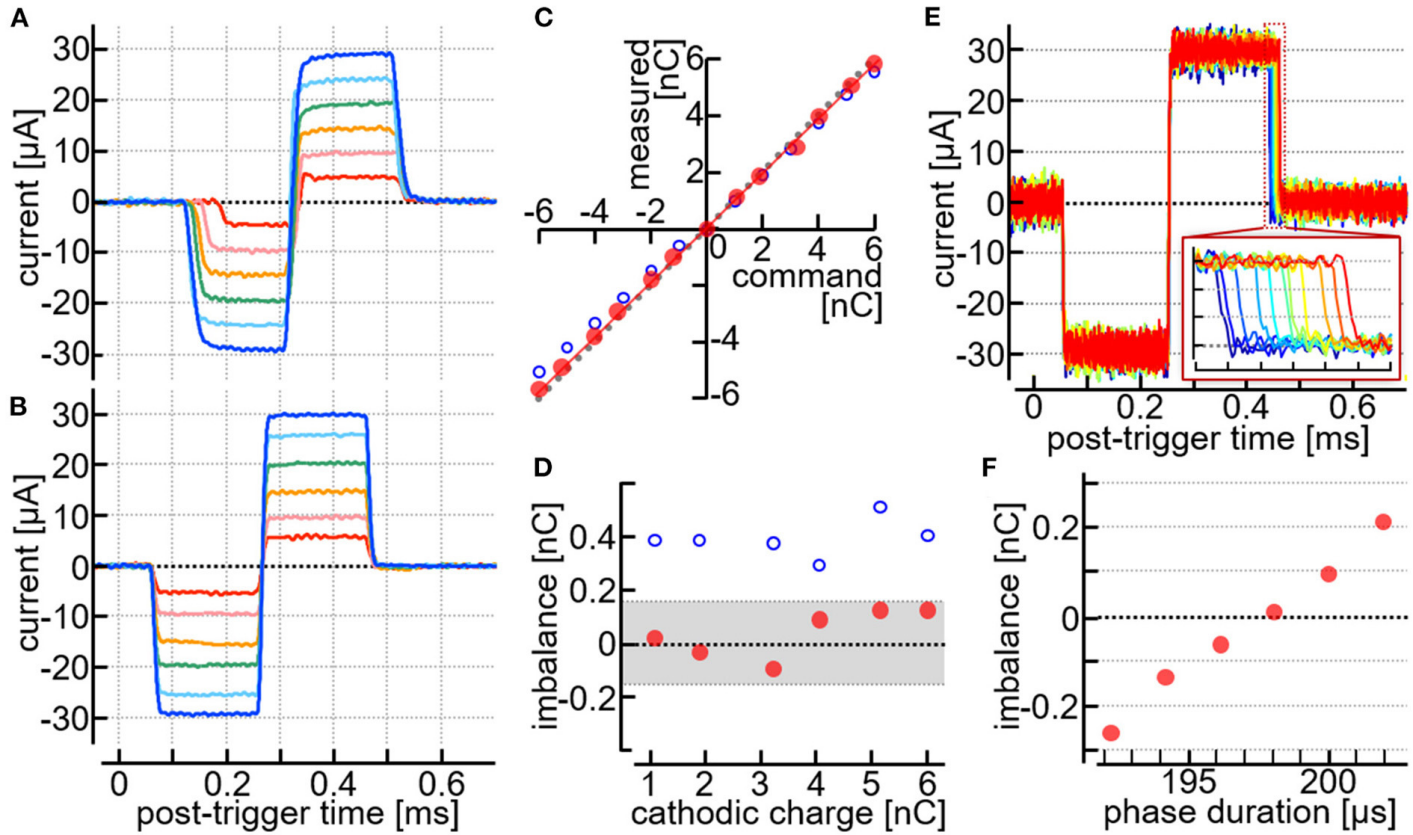
Stimulus current pulses recorded in animals *in vivo*. **(A,B)** Recorded traces of the current pulses generated by a desktop stimulator **(A)** and the stimulation module **(B)** with the same MEA in the same rat. **(C,D)** Measured current amplitudes plotted against the command amplitudes **(C)**, and the imbalance charges plotted against the charges delivered by the cathodic pulses **(D)**. Data taken from the results in **(A,B)**. **(E,F)** Testing the pulse width adjustments of the anodic phase. The animal was different from one used in **(A–D)**. **(E)** Current traces with different settings of the anodic pulse width. Inset in **(E)**, Expanded view of the offset of the anodic phase. **(F)** Imbalanced charges plotted against the command duration of the anodic phase. The imbalanced charges were calculated as the time integral of the recorded current trace from time zero to 0.5 ms.

Although proper operations of the stimulation module as an electronic device were verified in the dry-bench test (Section Dry-bench test), yet the tests in animals *in vivo* were considered essential since the electrode-electrolyte interface as well as other current-passing pathway in the biological tissues could exhibit non-linear electro-chemical properties (60). Therefore, the current pulse outputs through the MEA inserted in the cortex *in vivo* was first examined. In addition to the stimulation module, the desktop stimulator was used as a reference in the same animals and MEAs. Figures 5A,B shows an example of the current pluses recorded as voltage drops across a 1-kΩ resistor between one of the stimulating electrodes and an output channel of the desktop stimulator (A) or the stimulation module (B). The command amplitude of the current pulse was varied in the range from approximately ±5 to ±30 μA by a ~5-μA step. With a given amplitude setting, the current traces were averaged across sixteen recordings to reduce noise. As shown in Figure 5A, the onset of the cathodic pulse with relatively small amplitude settings was delayed, and the rise and fall time at the onset and offset of the current pulses was 20 μsec or longer. These characteristics of the current outputs were within the specifications of the stimulator used here, thereby ensuring the practically adequate configuration of the electrode placement and the current-passing pathway in the animal. When the stimulator was replaced with the stimulation module (Figure 5B), the rise and fall time at the current pulse onset and offset was nearly equal to or shorter than 5 μsec, and the amplitude was kept stable in either the cathodic or anodic phase with every amplitude setting. This was similar also in other animals and electrodes (see Figure 5E and Supplementary Figures 2A,C), indicating the reliable control of stimuli by the module.

Figure 5C shows a plot of the charges delivered by the stimulus pulses in either the cathodic or the anodic phase. The measured values (i.e., the time-integral of the current trace) deviated slightly from the command values when the stimulation module was used (red dots in the plot). These deviations were similar between the negative and positive charges. Figure 5D shows the difference between the positive and negative charges (i.e., the imbalanced charge) plotted against the charge delivered by the cathodic pulse. Since the maximum output range of the module was set to be ~±100 μA and in turn the amplitude resolution was approximately 1.56 μA (see Section Design), the theoretical upper limit of the charge imbalance between the cathodic and anodic phases was ±156 pC. Consistently, the imbalanced charges (red dots in the plot) were smaller than this limit (indicated by hatching in the figure). Such a measurable amount of the charge imbalance could be reduced by utilizing the pulse-width adjustment in the stimulation module (see Section Design), as shown in Figures 5E,F. The current amplitude and the pulse duration were set to be ~±30 μA/phase and 0.2 msec/phase, respectively, as the command values. With these settings, the offset timing of the anodic phase was shifted from one to the next by using the pulse-width adjustment function, and the corresponding current traces were recorded, as shown in Figure 5E. In the figure, eleven traces are superimposed, and each of these traces represents a single recording sweep. The inset shows an expanded view of the current traces near the offset of the anodic phase. Figure 5F plots the imbalanced charge against the adjusted pulse width of the anodic phase. At the best setting in this example, namely a pulse width shorter than 200 μsec by ~1.95 μsec, the imbalanced charge was reduced marginally below +6 pC.

**Figure 6:**
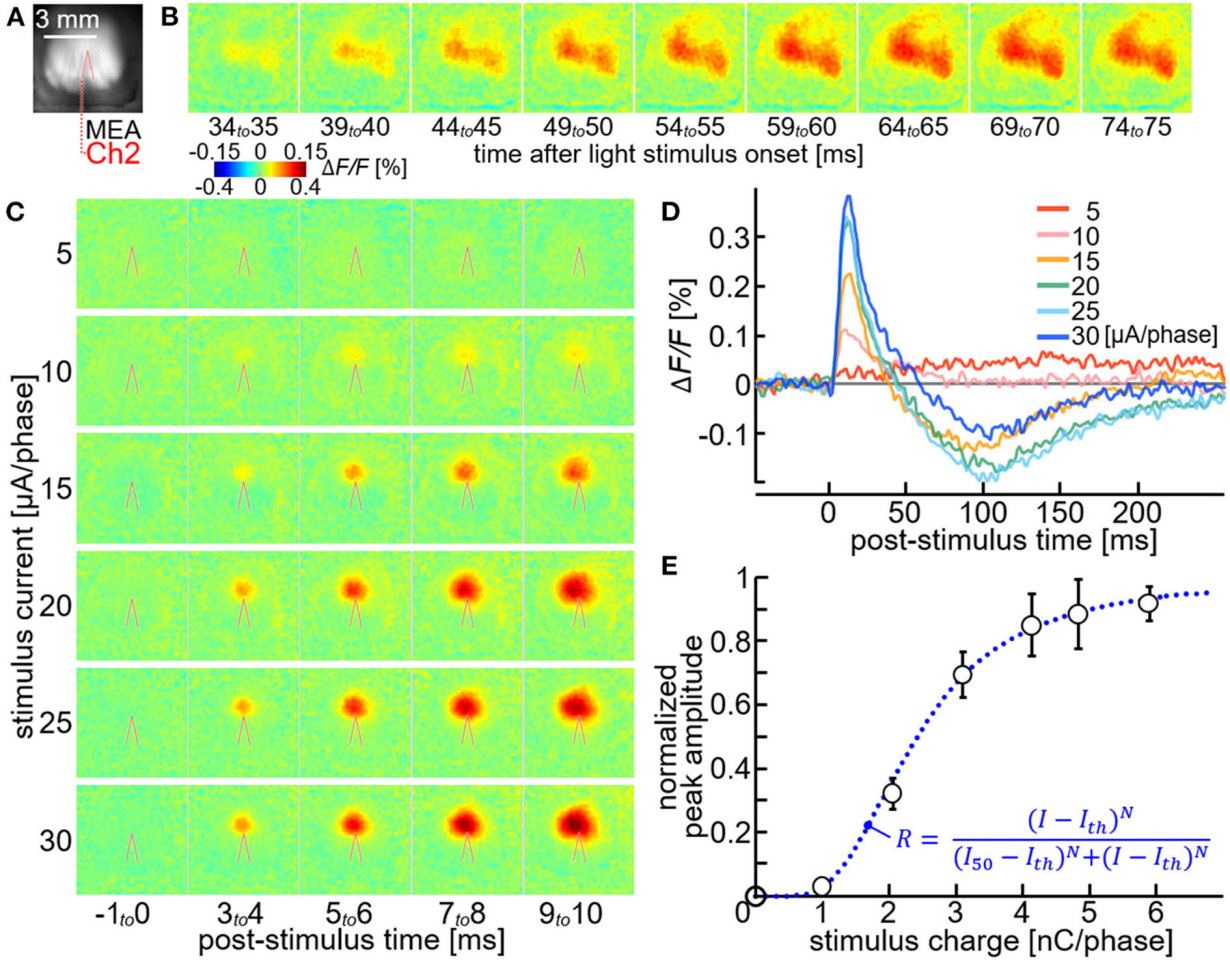
Neural excitations with using the stimulation module. **(A)** VSD fluorescence image of the cortex. Red line indicates the outline of shadow of the stimulating electrode shaft. **(B,C)** Pseudo-color time-lapse images of the rational VSD fluorescence change (Δ*F/F*) induced by the light stimulus **(B)** or the microstimulation with different current amplitudes **(C)**. **(D)** Time courses of the Δ*F/F* measured near the stimulating electrode tip. Different colors represent different stimulus amplitudes. **(E)** Normalized peak response amplitude plotted against the stimulus charge. The data were obtained with a total of 8 electrodes in 6 rats. The plot and error bars indicate the mean ± s.e.m. for the 8 data sets. The smooth line indicates the fitted curve of a modified Hill function (inset).

Finally, the usability of the stimulation module for the physiological experiments *in vivo* was examined. The same set of the current pulses shown in Figure 5 was used as the stimuli. Figure 6C shows an example of pseudo-color time-lapse images of the Δ*F/F* signal in response to the stimuli delivered *via* one of the electrodes in MEA. As compared in the top two rows of the figure, when the current amplitude was greater than ~5 μA/phase, the increase in Δ*F/F* corresponding to neural excitations appeared significant. The increase in Δ*F/F* was initially induced near the tip of the stimulating electrode (red line in the figure shows the outline of shadow of the electrode), and then propagated to its surrounding area in the next several milliseconds. The spatial extent of this propagation was larger as the current amplitude was increased from ~10 to ~20 μA/phase (the third and fourth rows from the top in the figure) and nearly saturated when the current amplitude was greater than ~20 μA/phase (two rows at the bottom in the figure). The saturation of the response was not due to a saturation of the output voltage in the stimulation module (Supplementary Figures 2A,B), and was similar when the desktop stimulator instead of the stimulation module was used in this particular animal and the electrode (Supplementary Figures 2C–F). Figure 6D shows the time courses of the changes in Δ*F/F* near the tip of the electrode. In response to the stimuli, the Δ*F/F* signal increased to reach the positive peak at 11–15 msec post-stimulus time, and then returned to the original level after a few to several hundred milliseconds. In the case that the current amplitude was greater than ~10 μA/phase, the Δ*F/F* signal decreased below the original level to reach the negative peak at around 100 msec post-stimulus time. These were consistent with those observed in the previous experiments (26, 27). Figure 6E plots the positive peak amplitudes of the Δ*F/F* responses against the stimulus charges. For this figure, the data sets obtained from multiple samples were averaged (*n* = 8 electrodes in 6 rats) and fitted with a modified Hill function (dotted blue line), providing approximate values of ~0.9 nC/phase for the threshold charge (*I*_*th*_), ~2.4 nC/phase for the charge that induced the half-maximum response amplitude (*I*_50_), and ~2.5 for the Hill coefficient (*N*).

**Figure 7:**
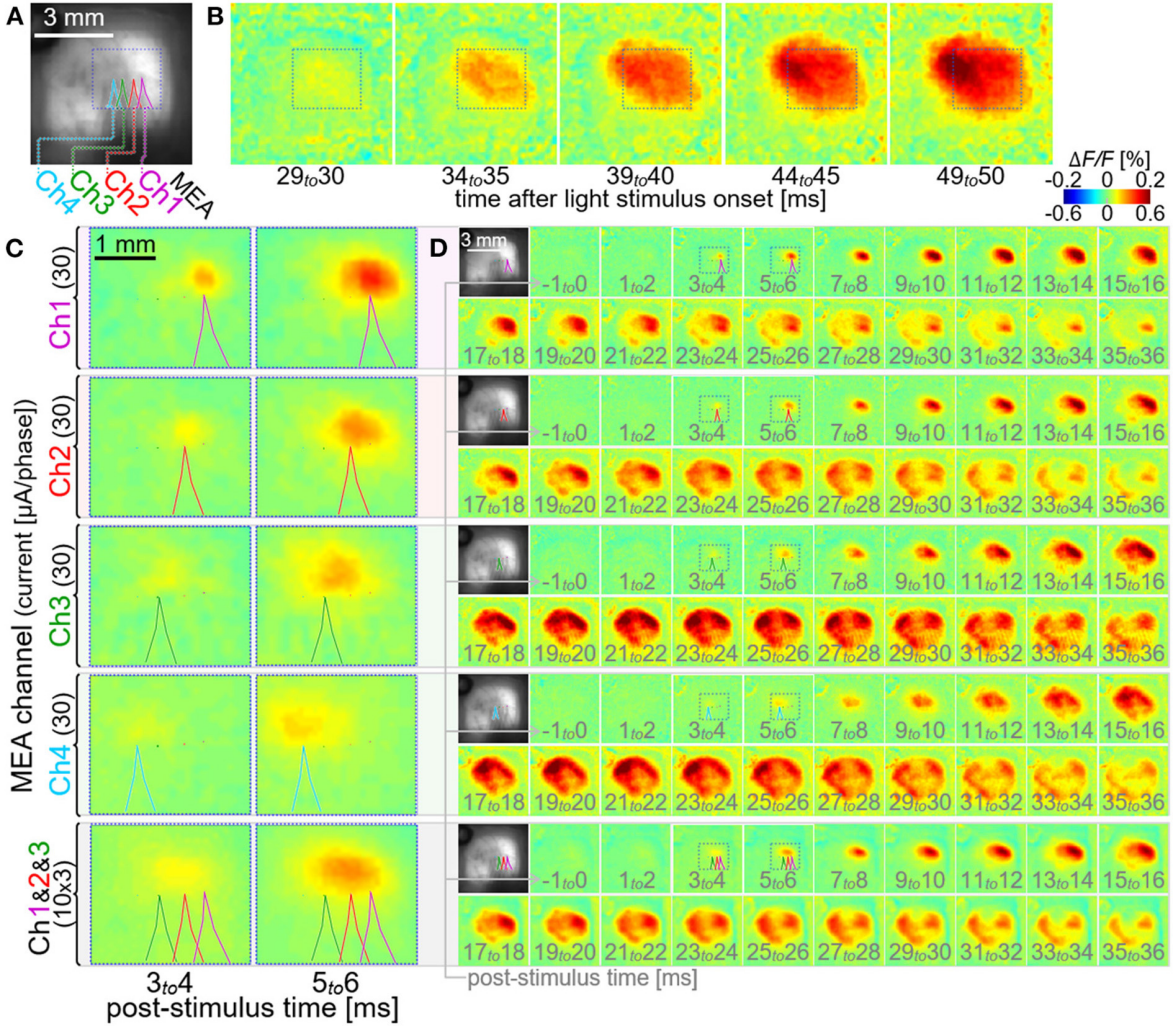
Multi-site neural excitations with using the stimulation module. **(A)** VSD fluorescence image of the cortex. Colored lines indicate the outlines of shadows of four stimulating electrode shafts, Ch1, Ch2, Ch3, and Ch4. **(B–D)** Pseudo-color time-lapse Δ*F/F* images induced by the light stimulus **(B)** or the current stimuli delivered individually via either one of the four electrodes [upper four rows in **(C,D)**] and simultaneously *via* three electrodes [bottom row in **(C,D)**]. The images in **C** show expanded views of the VSD images in the dotted boxes in **(D)**. All the dotted boxes in **(A–D)** represent the same region on the cortex in the animal.

Figure 7 shows an example of the multi-site neural excitations with using the stimulation module. Figure 7A shows the VSD fluorescence image of the exposed cortex superimposed with the locations of four electrodes of the MEA (colored outlines of shadows of the electrodes; Ch1, Ch2, Ch3, and Ch4). The current pulse with the amplitude of ~30 μA/phase was delivered from either one of those four electrodes (four rows from the top in Figures 7C,D). In each of the four cases, the increase in Δ*F/F* was initiated at around a few milliseconds post-stimulus time, as shown in Figure 7C. The center peak positions of these initial responses shifted from one another by around 0.3–0.4 mm within the V1, consistent with the tip positions of the four electrodes (outlines of shadows of the electrodes). In each of the four cases, the increase in Δ*F/F* propagated to its surrounding area in V1 in the next several milliseconds, and then further circuit activations were induced in V1 as well as extra-V1 areas in the next tens of millisecond, as shown in Figure 7D. The spatiotemporal patterns of those cortical activations differed from each other among the four cases. In the experiment shown in the bottom row in Figures 7C,D, the current pulses with the amplitude of ~10 μA/phase were delivered simultaneously from three of those electrodes (Ch1 to 3). As shown in the image frames at 3–4 and 5–6 msec post-stimulus time (Figure 7C), the spatial profile of the initial increase in Δ*F/F* was slightly more elongated in the anterior-posterior direction than those induced by the single-site stimulation. The spatiotemporal pattern of the cortical activation induced after this initial excitation appeared somehow similar to that induced by the stimulus with the electrode Ch2 (Figure 7D).

**Figure 8:**
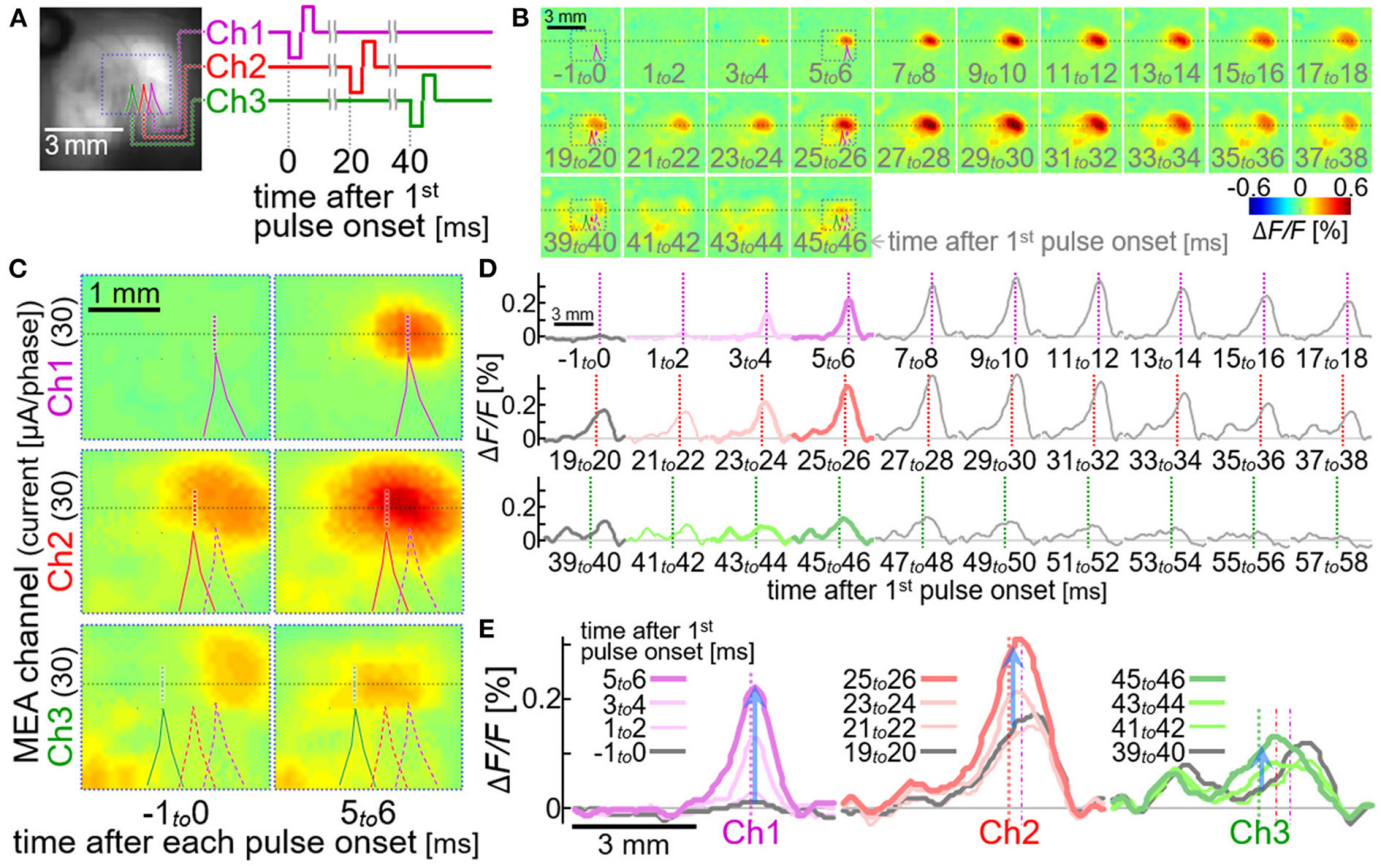
Sequential multi-site neural excitations with using the stimulation module. **(A)** VSD fluorescence image of the cortex. Colored lines indicate the outlines of shadows of three stimulating electrode shafts, Ch1, Ch2 and Ch3. The diagram on the right shows timing of the stimulus pulses delivered from the three electrodes. **(B,C)** Pseudo-color time-lapse Δ*F/F* images induced by the sequential stimulation. The images in **(C)** show expanded views of the VSD images in the dotted boxes in **(B)**. All the dotted boxes in **(A–C)** represent the same region on the cortex in the animal. **(D,E)** Spatial line profiles of the VSD signal along the line in **(B)**. Colored profiles in **(D)** are expanded and superimposed in **(E)**. Upward blue arrows represent the positions and the amplitudes of the maximum increases in VSD signal induced in 5–6 msec after the stimulus pulses.

Figure 8 shows an example of the sequential multi-site neural excitations with the stimulation module. The current pulse with the amplitude of ~30 μA/phase was sequentially delivered from the electrode Ch1 to Ch 3. The time interval between the two pulses was set to be 20 msec (Figure 8A), since the previous physiological study showed that the inhibitory circuit is recruited in this time range after the preceding stimulus pulse (27, 61). As expected, the circuit activation seen after the second stimulus pulse decayed more rapidly (VSD images and spatial line profiles from 29 to 38 msec in Figures 8B,D, respectively) than that seen after the first stimulus pulse (VSD images and spatial line profiles from 9 to 18 msec in Figures 8B,D, respectively), and the neural excitation induced by the third stimulus pulse (bottom row in Figure 8C; green traces in Figures 8D,E) was significantly smaller in amplitude than those induced by the first and second stimulus pulses (top and middle rows in Figure 8C; purple and red traces in Figures 8D,E).

To our best knowledge, this is the first time that the spatio-temporal patterns of cortical neural responses to multi-channel stimulation at a millisecond time resolution was examined. The results demonstrated that the stimulation module can be used to induce the neural excitations in different spatial locations within V1, and to examine the non-linear interaction of those neural excitations and the subsequent circuit activations in the visual cortices. Further experiments are necessary to investigate quantitative relationships between the spatiotemporal patterns of the stimulation and the corresponding neural responses. Nevertheless, these results suggested that the stimulation module is useful for such physiological investigations.

**Figure 9:**
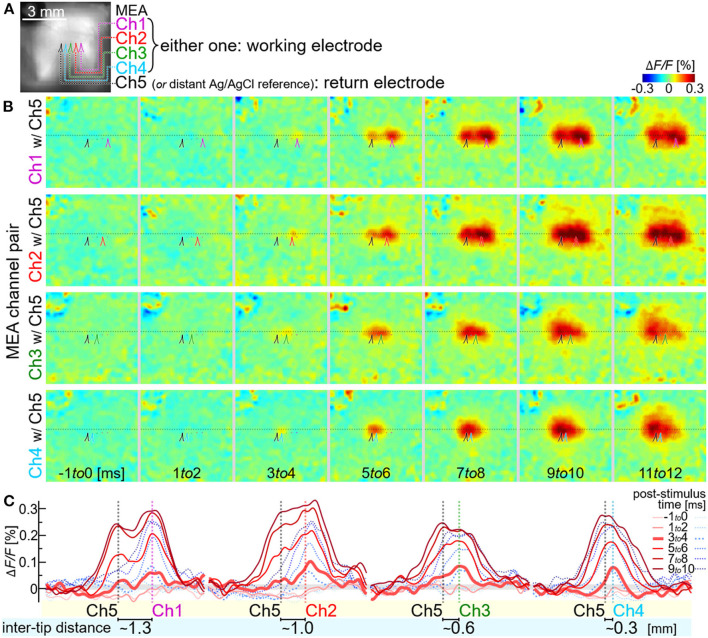
Neural excitations by the bipolar stimulation with using the stimulation module. **(A)** VSD fluorescence image of the cortex. Colored lines indicate the outlines of shadows of five stimulating electrode shafts, Ch1-Ch5. **(B)** Pseudo-color time-lapse Δ*F/F* images induced by the stimuli with different pairs of the electrodes. **(C)** Spatial line profiles of the VSD signal along the line in **(B)**. Red and blue traces indicate the responses to the bipolar and the monopolar stimulations, respectively. Either of the working electrodes, Ch1 to Ch4, and the distant Ag/AgCl reference electrode were used for the monopolar stimulation.

We also examined the neural excitations induced by the bipolar stimulation (Figure 9). One of five MEA channels (Ch5) was electrically shorted to the Ag/AgCl reference electrode by the switch in the output circuit (see boxes in the rightmost of Figure 1C) to act as a return electrode for stimulation. Either one of the remaining four MEA channels (from Ch1 to Ch4) were paired with this return electrode to act as a working electrode for stimulation. The current pulse with the amplitude of ~30 μA/phase was delivered from either of those four pairs of electrodes. The same current pulse was also delivered either of the four working electrodes in the monopolar configuration. Figure 9B shows the VSD images in response to the bipolar stimulation, and Figure 9C compares the spatial line profiles of VSD signals in response to the bipolar stimulation (red traces) and the monopolar stimulation (blue dotted traces). When the inter-tip distance of the MEA pair was ~0.6 mm or longer (i.e., when either of Ch1, Ch2 and Ch3 was used as the working electrode), the neural excitations were initiated in two discernible locations (3–4 msec post-stimulus time in Figure 9B), and those were larger in amplitude and wider in spatial extent (red traces in Figure 9C) than those induced by the monopolar stimulation (blue traces in Figure 9C). When the inter-tip distance of the MEA pair was ~0.3 mm (i.e., when Ch4 was used as the working electrode), the spatial profile of the neural excitation was similar to that induced by the monopolar stimulation (the rightmost plots in Figure 9C).

This result demonstrated that the ability of the stimulation module to arrange the working-returning electrode combination by electronic means can be beneficial in modifying the spatial pattern of neural excitation while keeping the stimuli the same.

## Dynamic operation test

**Figure 10:**
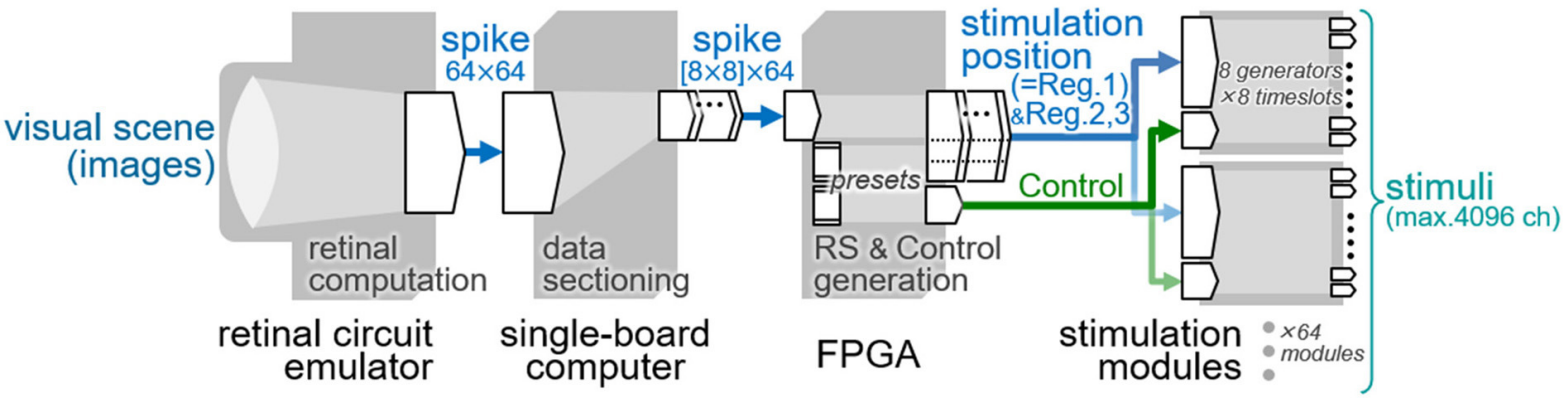
Block diagram of the testing system of the dynamic operation. The data flow from the left to right.

For future behavioral assessment of the phosphene-based vision in animals, a system capable of generating spatially patterned stimuli in accordance with the visual scene would be required (62). The present stimulation module was designed to generate a spatial pattern of stimuli with the 64 output channels based on the stimulation position data stored in the register, Reg. 1 in Table 1 (Section Design). Thus, by updating these data at a certain temporal rate, various stimulus patterns can be generated from moment to moment. In this section, such a dynamic operation with the stimulation module was tested by employing the previously developed retinal circuit emulator (63) as a data generator of the stimulation position. Figure 10 illustrates a block diagram of the testing system. First, the emulator (Figure 10, leftmost) captures and processes the incoming images to send out an image stream of the point-process spike signals, as describe previously (63). For the present system, the spike signal image is reformatted into 64-by-64 pixels at a time step of 5 msec [cf. 128-by-128 pixels, 0.5-msec resolution in the original format (63)]. Each pixel in the image takes a value of 0 or 1 and represents the all-or-none spike. Second, every frame in the image stream is sectioned into 64 blocks of 8-by-8 pixel by using a single-board computer (Raspberry Pi 4 Model B, Raspberry Pi Foundation, Cambridge, U.K.), and is sent block by block to the FPGA module. Third, each of those blocks is tagged with a particular Chip ID code by the FPGA, and is used as the stimulation position data for the particular stimulation module. The RS signal other than the stimulation position data (i.e., data for Reg. 2 and Reg. 3 in Table 1) as well as the Control signal are also generated by the FPGA. Fourth, all the settings (i.e., Reg. 1, 2 and 3) are stored in the built-in registers of the stimulation modules, and then the stimuli were output from those modules based on the Control signals. In the present, an 8-by-8 light-emitting diode (LED) array instead of stimulating electrodes is connected to the output channels of the stimulation module for a demonstration purpose. Each of the current generators in the stimulation module injects a monophasic current pulse (50 μA in amplitude, 1.8 msec in duration) into a driver circuit of each LED. Thus, the light of each LED is turned on or off depending on the 0 or 1 in the stimulation position data, and in turn, spatial stimulus patterns generated by the stimulation module are visualized as LED lighting patterns.

**Figure 11:**
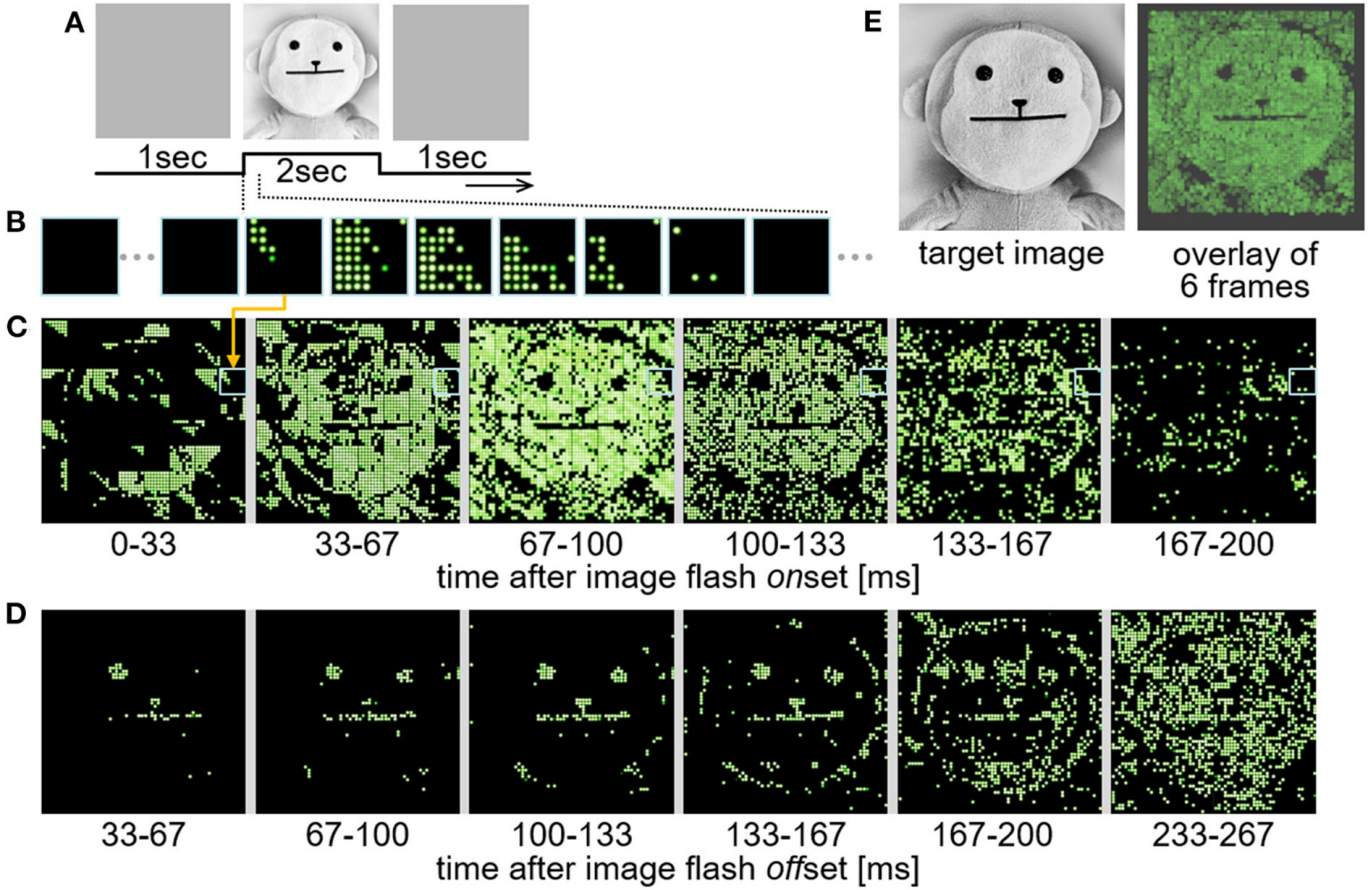
Demonstration test of the real time dynamic operation. **(A)** Image flash stimulation applied to the retinal circuit emulator. An image of a doll was displayed for 2 seconds after a full-field gray image. **(B)** An example of time-lapse images of the 8-by-8 LED lighting patterns generated by the current outputs from the stimulation module. Each of these images are digital photographs captured at ~30 fps by a camcorder. **(C,D)** Combined time-lapse images of 64 of the 8-by-8 LED lighting patterns generated by the stimulation module after the onset **(C)** and the offset **(D)** of the image flash stimulation. **(E)** Comparison between the target image (left) and the resulting LED lighting pattern (right) in which 6 image frames shown in **(C)** are overlaid.

In the test using this system, the input images (Figure 11A) were displayed on a computer monitor in front of the retinal circuit emulator and the resulting LED lighting patterns (Figure 11B) were captured by a camcorder (EX-ZR1800, Casio Computer Co., Ltd., Tokyo, Japan). The frame rates of the monitor and the camcorder were 144 and 30 frame-per-second, respectively. In the present, one pair of the stimulation module and the LED array was used to save hardware resources and avoid any complications in the test. Although the FPGA in the system was capable of feeding all the stimulation position data to the 64 stimulation modules at a certain frame rate, only one of those data blocks was actually used for the current outputs from the stimulation module (the other data blocks were discarded). Therefore, a total of 64 trials were performed while changing the block used for the current outputs, and in each of those trials, the LED lighting patterns were captured as an image stream by the camcorder. The 64 image streams were combined offline. Figures 11C,D shows an example of the time-lapse images taken from the combined image stream. In this test, the output response type emulated in the retinal circuit emulator was a transient ON type, which generates spikes in response to positive contrast steps in an image stream (63). Hence, the LEDs were turned on by the positive contrast steps appeared at the onset and offset (Figures 11C,D, respectively) of the image flash stimulation shown in Figure 11A. For a better comparison with the image flash stimulation, the 6 image frames shown in Figure 11C are overlaid in Figure 11E (right panel), showing a reasonable correspondence between the target image (left panel) and the resulting LED lighting pattern. Here, the rate of updating the spatial stimulus pattern with the 4,096 channels was around 30 frame-per-second. This limit of the data transmission was due to the transmission rate from the single-board computer to the FPGA. Nevertheless, this result showed that the present stimulation module is applicable to a dynamic pattern stimulation in accordance with the visual scene.

The hardware volume of the 64-channel system used in this test was about 22.5 × 7 × 11.5 (w×h×d) cm without the retinal circuit emulator (Supplementary Figure 3). To extend this to a 4,096-channel system, 64 boards of the stimulation module (“d” in Supplementary Figure 3) and 8 printed-circuit boards that connect the interface circuit board (“c” in Supplementary Figure 3) to the stimulation module boards are needed. These components are estimated to consume a volume of about 10 × 4 × 4.5 (w×h×d) cm. Therefore, the total hardware volume of the 4,096-channel system is estimated to be 22.5 × 7 × 14 (w×h× d) cm or smaller. The power consumption of the 64-channel system without the emulator was around 9.6 W or lower in total, including ~4–7 W for the single board computer, ~2.0–2.5 W for the FPGA module, ~3.6 mW for the stimulation module (see Section Dry-bench test), and others. Although the exact amount of the power consumption varies with time and depends on the state, the total power consumption of the 4,096-channel system is estimated to be around 9.83 W or lower. Based on these estimates, the 4,096-channel system is considered to be practically usable for experimental studies in restrained animals, but difficult in freely-moving animals. In order to use the 4,096-channel system in freely-moving animals, wireless data and power transmission devices together with data compression/expansion processors are thought to be required.

## Discussion

In this study, we developed the multichannel stimulation module for pre-clinical animal experiments (Figure 1). The proper operations of this module were verified in the dry-bench tests (Figures 2, 3) and with the intracortical electrodes in experimental animals *in vivo* (Figures 4, 5). Practical usefulness of this module for the physiological investigations in experimental animals *in vivo* was demonstrated (Figures 6–9). Also, we showed that the dynamic operation of our stimulation module enables the spatial patterns of stimuli can be updated at a certain frame rate in synchronization with the visual scene (Figures 10, 11). For induction of spatially discernible multiple phosphenes in the visual filed, the electrodes delivering the stimuli in the cortex should be separated from each other at certain distances. In clinical trials on intracortical stimulation of the visual cortex in humans (14, 21) and in psychophysical studies in non-human primates (8, 31), the shortest distance between a pair of adjacent electrodes delivering the threshold stimuli to induce two distinct phosphenes was estimated to be around 0.5 mm (64). Assuming that the exposed surface area of the hemispheric V1 (not including the area in the calcarine fissure) is around 689 mm^2^ (65), the total number of the electrodes implantable in this area is approximately 2,700 if they are placed at the shortest inter-electrode distance (i.e., 0.5 mm). Such high-density electrode implantation might be infeasible considering the difficulties of the surgery of the electrode insertion (66). A possible alternative strategy is to implant the electrodes at relatively longer inter-electrode distances (e.g., 1-2 mm) in the area of not only V1 but also the secondary visual cortex (2) in both hemispheres such that phosphenes might be induced in a relatively large portion of the visual field (34, 35). With either possibility, the number of the electrodes achievable in the future is thought to be in the range of a few to several thousand channels. The recent experiments in non-human primates have suggested the feasibility of a high-channel-count stimulation to achieve the phosphene-based shape perception (62). For designing the effective stimuli with using such a large number of stimulation channels, unraveling the physiological basis of the phosphene induction through preclinical animal experiments is thought to be inevitable.

A microstimulation system with the fully distributed architecture (41) and monolithic architecture (67) capable of driving nearly or more than a thousand intracortical electrodes have been previously proposed. With such a large number of the channels, the spatial pattern of stimulus should be updated dynamically in response to the visual scene of the outside world, although the transcutaneous wireless data transmission for the implant are limited (45, 46). Therefore, in order to achieve a certain frame rate for updating the stimulus pattern, a data compression/expansion function implemented in micro-processors would be required for future prosthetic systems (68, 69). In envisioning such a system with embedded micro-processors (68), the modular architecture is thought to be preferable for control of the stimuli with a large number of the electrodes in a unified manner. In previous, a modular architecture system capable of supporting nearly one thousand stimulating electrodes has been developed (36). Since this system was designed specifically for clinical application purpose, the microstimulation module chip included in the system had the output pads that could be directly attached to a multielectrode array of a specific design (a 4-by-4 array of electrodes with inter-electrode distance of 0.4 mm). Also, in other multi-channel stimulation devices designed for the clinical application of the cortical visual prostheses, the stimulation device and the multi-electrode array are designed to be packaged together (40, 70). Therefore, with either of those devices, the spatial arrangement of the electrodes is fixed, and not adjustable to various experiments in animals. The difference in design between the devices for the clinical application and the animal experiment is thought to be based on a priority trade-off between the safeness and the multi-functionality (flexibility) since the floor space and affordable energy consumption in a device chip is limited. One of the most important concerns in clinical use of a multi-channel stimulator may be the safeness of stimulation in long-term use. For this objective, the on-chip wireless communication function, which eliminates the need for the troublesome (possibly unsafe) hardwiring from a stimulator device to the electrodes, would be feasible (40) [see also Wong et al. (70)]. However, implementing this function in a chip may not allow many output channels and/or other (possibly redundant) functions to be implemented in the same chip to save the floor space and energy consumption. On the other hand, the stimulator device that is usable for animal experiments may require multiple functions and scalability, with which physiological/behavioral effects of a variety of stimulus parameters can be examined in different animal models with different cortical sizes. In the present stimulator module, one of the key features that differentiate it from the previously proposed stimulators is the combination of various electronic functions. Those include the on-chip register memory, the pulse width adjustment, the output circuit in every channel, and the chip ID elements for the common-bus digital control. By using these functions, not only the shape of each biphasic current pulse (i.e., the cathodic/anodic polarity order, the amplitudes and durations in cathodic and anodic phases, and the inter-phase intervals) but also other parameters (e.g., the degree of charge imbalance, the combination of working-returning electrodes, the temporal order of pulse deliveries among multiple channels), as well as the number of stimulating electrodes in operation can be digitally controlled in a dynamic way. Besides, different from the previous devices, the accurate controls of stimuli by the present module have been proven under the practical conditions of animal experiments. We would not claim that the present stimulation module is state-of-the-art in terms of electronic design, but we do believe that our module is a proven electronic device useful in animal experiments.

During injection of the stimulus current *via* the electrode, the voltage drop at the electrode-electrolyte interface must be maintained within the water potential window in order to prevent the water electrolysis and irreversible reactions of the electrode material (11, 12). Previous studies suggested that some types of the metal-surface electrodes meet such a requirement (71–73) and are considered to be usable for intracortical stimulation (14, 37, 38, 74). The potential window with those electrodes is roughly from −1 to +1 V (11). Thus, although the limit voltage of ~±2.4 V in the present stimulation module can be a limiting factor of the stimulus current amplitude (Figures 2A,B,F,G), the water potential window is more critical than this limit voltage in the intracortical stimulation. We used the electrodes with activated iridium-oxide surface in our experiments (Figures 4B–D) and verified that the interface voltage drop could be maintained below the safe limit (11, 12) while the neural excitations were induced (Supplementary Figure 2). We also observed that the interface voltage was asymmetry in waveform between the cathodic and anodic phases (Supplementary Figures 2B,D), consistent with the asymmetric nature of the chemical reactions underlying the charge deliveries between these opposite phases, and with previous observations made in saline *in vitro* (11). Considering the non-linearities of both charge injection and neuronal excitability, it would be interesting to examine the effects of asymmetric current pulses on the electrode-electrolyte interface voltage and the neuronal membrane voltage at high temporal resolution. For such electrochemical and physiological experiments, the present stimulation module can offer reliable control of the stimulus waveform (Figure 5). In addition, the stimulation module can generate the stimulus current greater than ±100 μA/phase in amplitude (Supplementary Figure 1). It is estimated that the impedance of an Ir-Ox-surface electrode with a conical tip of ~64 μm in base diameter and ~320 μm in length should suffice for delivering the stimulus current of 100–400 μA/phase in amplitude while keeping the voltage within the water potential window. However, the insertion of such a large electrode in the cortex should induce the serious foreign-body tissue response in a few tens of minutes (75). In addition, the stimulation with such a large current may not be suitable for inducing a spatially localized neural excitation. Therefore, it is practically infeasible for one to deliver the large current to the neurons with an intracortical electrode in the acute physiological experiment. Another option is to use a planar electrode with a large diameter (e.g., ~200 μm, resulting an interface area similar to the above-mentioned large conical-tip electrode) placed on the cortical surface. The present stimulation module should be usable for such a cortical surface stimulation with the large current, although in this case, a different recording technique rather than the VSD imaging is needed for testing the effects of stimuli on neural responses.

Since we employed rats as the experimental animal and the optical imaging as the measurement method, the number of stimulating electrodes inserted in their V1 without significantly obscuring the cortical surface from sight under the microscope optics was limited to several, at least in the present experimental setup. Therefore, the stimulation patterns we examined were relatively simple, but not very elaborated in terms of electronics. Such simplicity, however, allowed us to analyze the correspondence between the stimuli and the resulting neural responses at fine spatiotemporal scales. In the present study, this is considered essential in confirming the physiological effects of microstimulation generated by the present stimulation module. Previously, the current steering/focusing technique was applied in cochlear implants (76) [also Constandinou et al. (77) for the vestibular prosthesis] and retinal prostheses (78). On the other hand, it is uncertain whether or how such a stimulation strategy is beneficial for the intracortical neural prostheses. This is in part since the neural responses to multi-site microstimulation in the cortex have not been revealed quantitatively through physiological experiments in previous studies. Based on the present experiments in the rat visual cortex *in vivo*, the spatial profile of the neural excitation appeared to be modifiable by the spatial arrangement of the stimulus current delivery *via* multiple intracortical electrodes (Figures 7, 9). A recent study proposed a novel type of multielectrode lead in which logically configurable 216 microelectrode pads are implemented (79). The ability of dynamically combining the microelectrode pads into several groups of the stimulus bus line in this state-of-the-art electrode device seems to be compatible with the time-division multiplexing current outputs in our stimulation device. Such a combination might open a new possibility of highly flexible neural stimulation in not only the deep brain stimulation, but also cortical prostheses.

Since the action potentials are initiated in a neuron population within a millisecond after the stimulus pulse onset (61), the VSD imaging at a millisecond resolution is considered to be suited for revealing dynamic interactions among stimuli and neural responses across multiple sites with the current steering/focusing stimuli in the visual cortices (27, 28, 56) as well as the somatosensory cortex (80). The multi-site intracortical stimulation generated by using our stimulation module and the VSD imaging may serve as a useful platform for examining the effects of different strategies of multi-site stimulation on the spatiotemporal neural responses. Although the VSD imaging can supply a foundation for testing the spatio-temporal neural responses to multi-site microstimulation, we also need to test/evaluate the perceptual consequences of the stimulations (81). Therefore, as a next step, the *in-vivo* physiological experiments should be combined with psychophysical/behavioral experiments in the same animal models for further understanding and development of cortical prostheses. As we showed in the demonstration test (Figure 11), multiple number of the stimulation module can be used in a unified manner for the dynamic pattern stimulation. Since the number of the module used is scalable for different animals with different cortical area where the electrodes are implanted, the different levels of the phosphene perceptions (e.g., from brightness localization to shape perception, or from static phosphene to moving phosphene) would be tested in those different animal models.

## Data availability statement

The original contributions presented in the study are included in the article/Supplementary material, further inquiries can be directed to the corresponding author.

## Ethics statement

The animal study was reviewed and approved by Animal Experiments Committee of Osaka University.

## Author contributions

TY and YH designed the study. YH, SK, and TY designed the specifications of stimulation module. SK designed the circuits of stimulation module. SK and YH conducted the dry-bench tests. YU and YH designed and conducted the animal experiments and analyzed the animal experimental data. SI and YH developed and tested the dynamic operation testing system. YH wrote the manuscript. All authors contributed to the article and approved the submitted version.

## Funding

This work was supported partly by the MEXT project, Creating Hybrid Organs of the future at Osaka University, and Grant-in-Aid for Scientific Research from MEXT, Japan (18K12059 and 22K12781 to YH and 20H00606 to TY).

## Conflict of interest

The authors declare that the research was conducted in the absence of any commercial or financial relationships that could be construed as a potential conflict of interest.

## Publisher's note

All claims expressed in this article are solely those of the authors and do not necessarily represent those of their affiliated organizations, or those of the publisher, the editors and the reviewers. Any product that may be evaluated in this article, or claim that may be made by its manufacturer, is not guaranteed or endorsed by the publisher.

## Supplementary material

Supplementary Figure 1 Example of the current outputs with different maximum amplitude settings in an output channel of the stimulation module. Panels from top to bottom rows show the recorded current traces as the 2-bit code of Max amplitude (see Table 1) was varied from 3 to 0 (in decimal number). In each panel, the 6-bit codes of Cathodic and Anodic phase amplitudes (see Table 1) were changed from 63 to 0 (in decimal number) along the time axis.

Supplementary Figure 2 Recorded traces of the stimulus current pulse **(A,C)** and the resulting output voltage **(B,D)** in the stimulation module **(A,B)** and the desktop stimulator **(C,D)**. **(E)** Pseudo-color time-lapse Δ*F/F* images induced by the stimuli with the desktop stimulator. **(F)** Time courses of the Δ*F/F* measured near the stimulating electrode tip. Different colors represent different stimulus amplitudes. The experiments here were made with the same electrode in the same animal as those in Figures 6A–D.

Supplementary Figure 3 A photograph of the 64-channel system used in the test shown in Figure 11.
