# Supplementary figures and images for: Multichannel stimulation module as a tool for animal studies on cortical neural prostheses
Source: Front Med Technol. 2022 Sep 13;4:927581. doi: 10.3389/fmedt.2022.927581 (PMC9513350; doi:10.3389/fmedt.2022.927581)

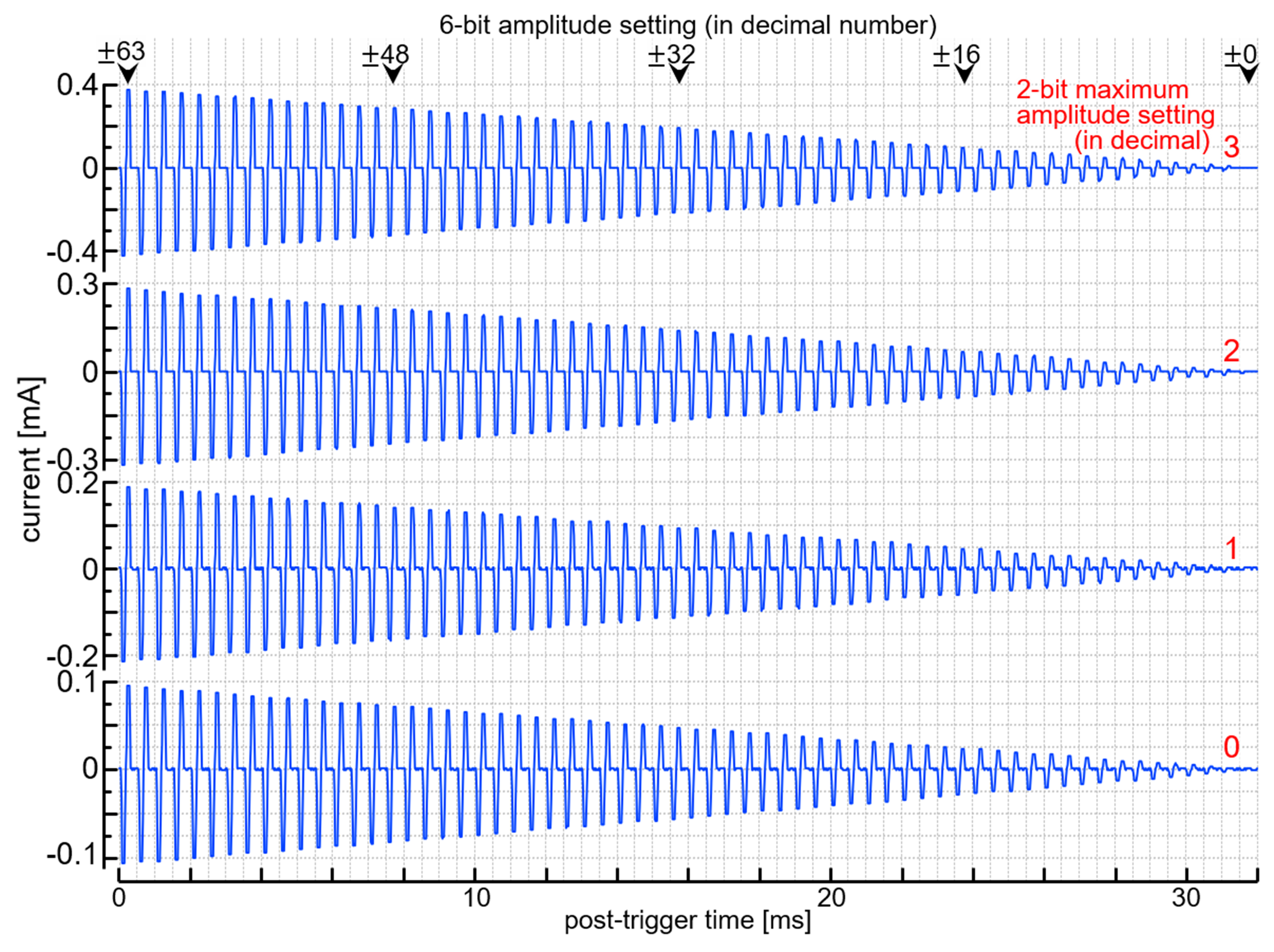

Supplement: Supplementary Figure 1 — Example of the current outputs with different maximum amplitude settings in an output channel of the stimulation module. Panels from top to bottom rows show the recorded current traces as the 2-bit code of Max amplitude (see Table 1) was varied from 3 to 0 (in decimal number). In each panel, the 6-bit codes of Cathodic and Anodic phase amplitudes (see Table 1) were changed from 63 to 0 (in decimal number) along the time axis. [file Image_1.TIF]

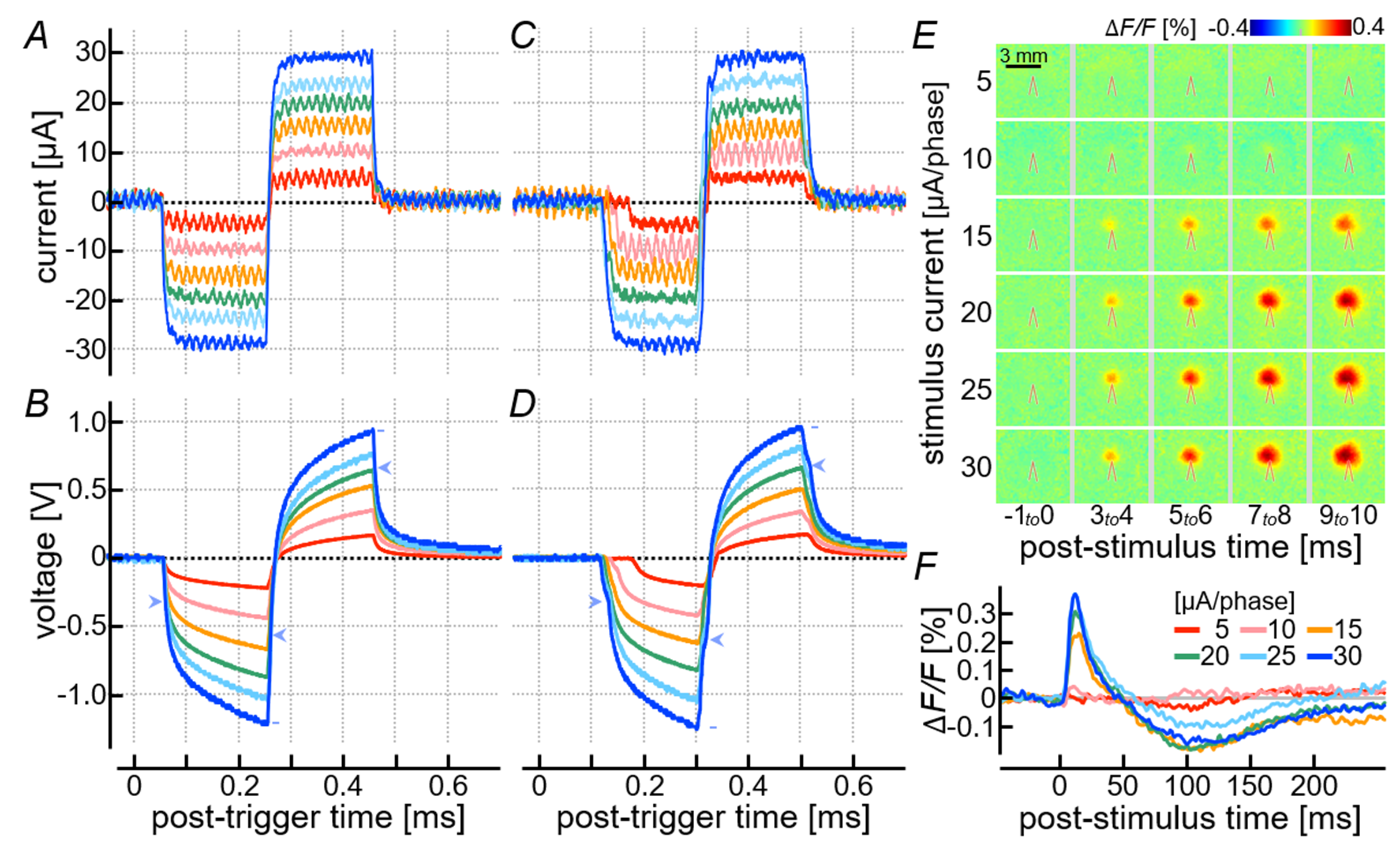

Supplement: Supplementary Figure 2 — Recorded traces of the stimulus current pulse (A,C) and the resulting output voltage (B,D) in the stimulation module (A,B) and the desktop stimulator (C,D). (E) Pseudo-color time-lapse ΔF/F images induced by the stimuli with the desktop stimulator. (F) Time courses of the ΔF/F measured near the stimulating electrode tip. Different colors represent different stimulus amplitudes. The experiments here were made with the same electrode in the same animal as those in Figures 6A–D. [file Image_2.TIF]

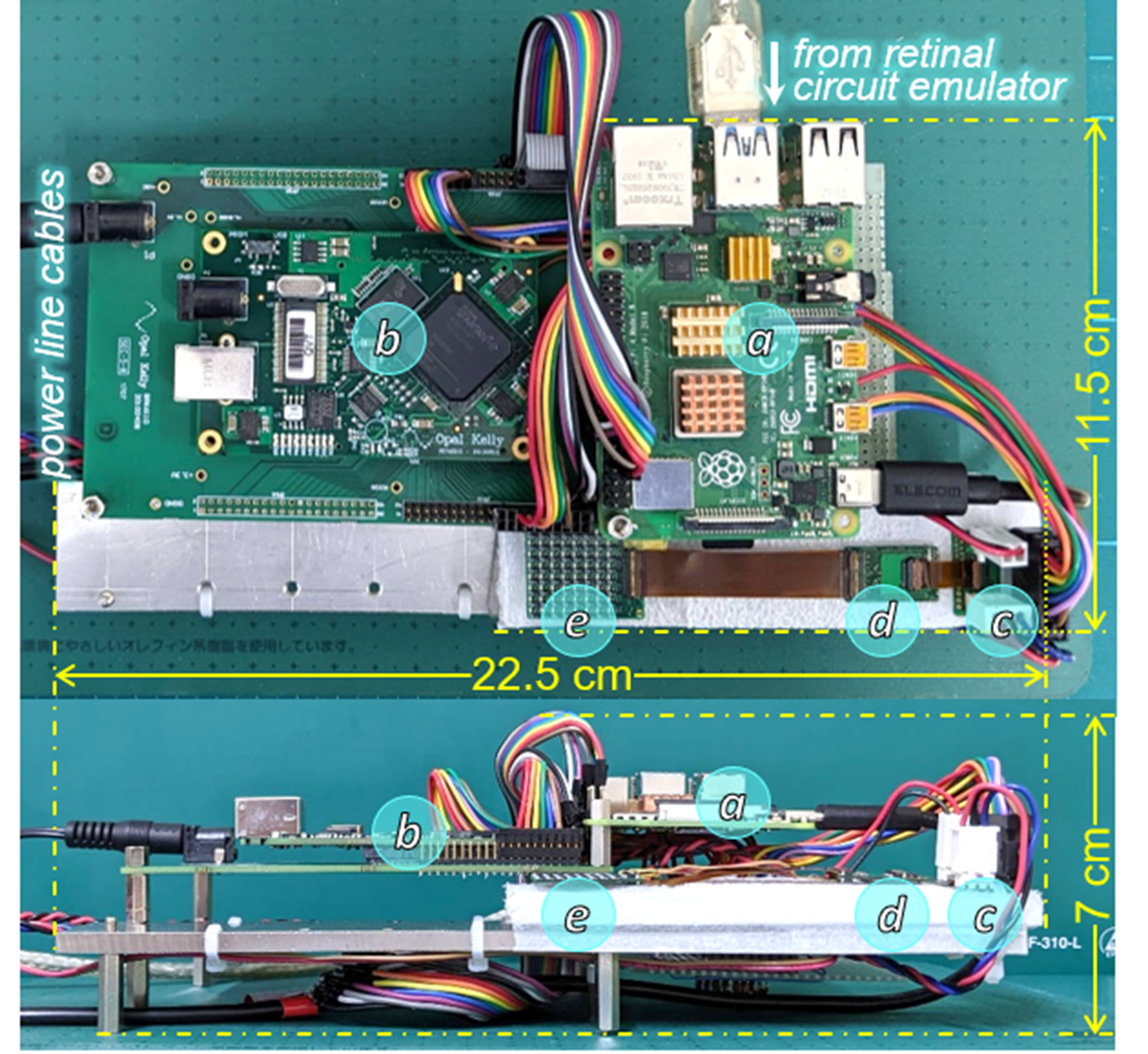

Supplement: Supplementary Figure 3 — A photograph of the 64-channel system used in the test shown in Figure 11. [file Image_3.TIF]
